# Design, Cytotoxicity and Antiproliferative Activity of 4-Amino-5-methyl-thieno[2,3-d]pyrimidine-6-carboxylates against MFC-7 and MDA-MB-231 Breast Cancer Cell Lines

**DOI:** 10.3390/molecules27103314

**Published:** 2022-05-21

**Authors:** Anelia Mavrova, Stephan Dimov, Inna Sulikovska, Denitsa Yancheva, Ivan Iliev, Iana Tsoneva, Galya Staneva, Biliana Nikolova

**Affiliations:** 1 Department of Organic Synthesis, University of Chemical Technology and Metallurgy, 8 Kliment Ohridski Blvd., 1756 Sofia, Bulgaria; anmav@abv.bg (A.M.); s_t_e_v_e@abv.bg (S.D.); 2 Institute of Experimental Morphology, Pathology and Anthropology with Museum, Bulgarian Academy of Sciences, Acad. G. Bonchev Str., bl.25, 1113 Sofia, Bulgaria; inna_sulikovska@ukr.net (I.S.); taparsky@abv.bg (I.I.); 3 Institute of Organic Chemistry with Centre of Phytochemistry, Bulgarian Academy of Sciences, Acad. G. Bonchev Str., bl. 9, 1113 Sofia, Bulgaria; denitsa.pantaleeva@orgchm.bas.bg; 4 Institute of Biophysics and Biomedical Engineering, Bulgarian Academy of Sciences, Acad. G. Bonchev Str., bl.21, 1113 Sofia, Bulgaria; itsoneva@bio21.bas.bg (I.T.); g_staneva@yahoo.com (G.S.)

**Keywords:** 4-amino-thienopyrimidines, cytotoxicity, phototoxicity, antiproliferation, molecular structure, structure–activity relationship

## Abstract

Novel 4-amino-thieno[2,3-d]pyrimidine-6-carboxylates substituted at the second position were prepared by cyclocondensation of 2-amino-3-cyano-thiophene and aryl nitriles in an acidic medium. The design of the target compounds was based on structural optimization. The derivatives thus obtained were tested in vitro against human and mouse cell lines. The examination of the compound effects on BLAB 3T3 and MFC-10A cells showed that they are safe, making them suitable for subsequent experiments to establish their antitumor activity. The photoirritancy factor of the compounds was calculated. Using the MTT test, the antiproliferative activity to MCF-10A, MCF-7 and MDA-MB-231 cell lines was estimated. The best antiproliferative effect in respect to the MCF-7 cell line revealed compound 2 with IC_50_ 4.3 ± 0.11 µg/mL (0.013 µM). The highest selective index with respect to MCF-7 cells was shown by compound **3** (SI = 19.3), and to MDA-MB-231 cells by compound **2** (SI = 3.7). Based on energy analysis, the most stable conformers were selected and optimized by means of density functional theory (DFT). Ligand efficiency, ligand lipophilicity efficiency and the physicochemical parameters of the target 4-amino-thienopyrimidines were determined. The data obtained indicated that the lead compound among the tested substances is compound **2**.

## 1. Introduction

Thienopyrimidines are purine bioisosters, and the use of their skeleton is a key factor in the process of creating new derivatives with predefined properties. Some of the generated thienopyrimidines are reported to possess a broad spectrum of biological activities, such as anti-inflammatory, analgesic, ulcerogenic and antimicrobial affects [1,2]. Other derivatives act as negative allosteric modulators of the dopamine D2 receptor, QcrB (ubiquinol-cytochrome c reductase cytochrome b subunit) inhibitors in *Mycobacterium tuberculosis* or as cytotoxic agents [1,2,3,4]. Thieno [2,3-d]pyrimidine compounds reveal cytotoxic effects via various mechanisms e.g., thymidylate synthase inhibitors [5,6,7].

Since the quinazolines’ inhibitory activity to various tyrosine kinase receptors was discovered, a number of compounds with thienopyrimidine scaffold in the structure have been synthesized in order to evaluate their potential effects towards the tyrosine kinase receptors (RTKs) [8]. The protein kinase enzymes (PTK) phosphorylate the structure of other proteins by chemical addition of a phosphate group, thus provoking a functional change of the target proteins and modifying the enzyme activity. The overexpression of PTK is usually associated with cell proliferation, which leads to the emergence of tumorigenic formations; therefore, their inhibition is an anticancer therapy target.

A number of 4-amino-tienopyrimidine analogues have been synthesized and evaluated for their inhibition efficacy towards B-Raf kinases. B-raf isoforms play a significant role in cell growth and survival, so the development of B-raf inhibitors could be used as an important approach for anticancer therapy [8,9,10]. The most common B-Raf mutation is the replacement of valine by glutamic acid at amino acid position 600 (V600E). The studied thienopyrimidines, containing the amino group at the fifth position, demonstrated inhibitory activities in the nanomolar range [9].

**Figure 1:**
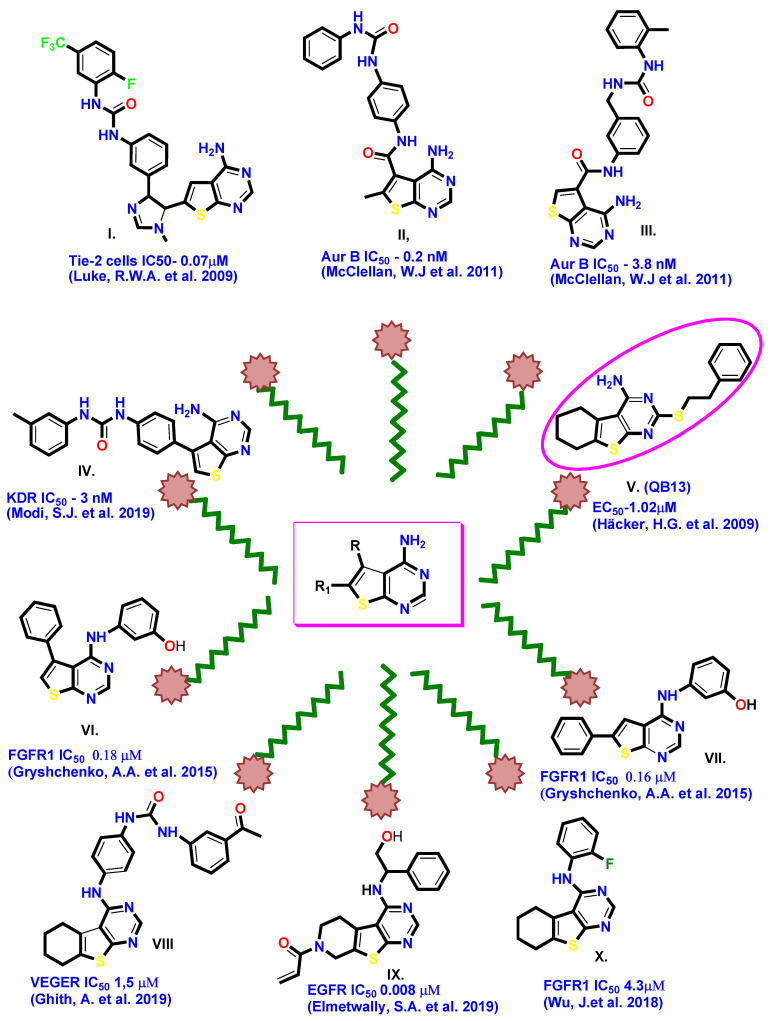
Reported thienopyrimidines as tyrosine kinases inhibitors [13,14,17,20,21,22,24,26].

Tie2 is another receptor tyrosine kinase participating in the angiogenesis of human tumors and is mainly expressed in the vascular endothelium. A novel class of 4-amino-thieno [2,3-d]pyrimidines, substituted at the sixth position, has been reported as potent inhibitors of Tie-2 [11,12]. One of the most active compounds possessing an IC_50_ value of 0.07 μM [13] is presented in Figure 1.

Aurora kinase members are potential therapeutic targets in cancer, as they play oncogenic roles related to their mitotic activity and promote cancer cell survival and proliferation. A number of potent and orally bioavailable thienopyrimidine derivatives have been generated as Aurora kinase inhibitors. The two most active compounds against Aurora B kinase showed IC_50_ values of 0.2 and 3.8 nM, respectively, as it is given in Figure 1 [14].

Epidermal growth factor receptor (EGFR) overexpression has been observed in many cancers, including breast cancer, lung cancer, colorectal and esophageal cancer [15,16]. Therefore, many 4-amino-thienopyrimidines with modified structures have been developed and their EGFR/HER2 inhibitory activities have been studied [17,18,19]. As it can be seen in Figure 1, the compound containing the fused piperidine ring (X) revealed IC_50_ in the nanomolar range [17]. A ligand-based design led to the synthesis of a series of 5-arylthieno [2,3-d]pyrimidines [19]. Their cytotoxic activity against the MCF-7 cell line and the inhibition of the enzymatic activity of EGFR-TK in vitro were investigated. Two of the studied 5-arylthieno [2,3-d]pyrimidines were reported to have cytotoxicity on the breast cancer cell line (MCF-7) with IC_50_ 9.1 nM and resp. 28.0 nM, and to show EGFR-TK enzyme inhibition at concentration of 1.83359 ng/mL and resp. 2.63671 ng/mL [19].

The fibroblast growth factor 1 receptor (FGFR1) is also related to the breast carcinogenesis [20]. A variety of novel thienopyrimidine derivatives have been synthesized as VEGFR-inhibitors. The VEGF, a protein with vascular permeability activity and its receptor (VEGFR) take place in the most pathological angiogenesis such as cancer. They regulate both vasculogenesis and angiogenesis of blood vessels [21,22,23,24]. 5-Arylthieno [2,3-d]pyrimidines were reported to show high FGFR1 inhibitory activity.

As it can be seen in Figure 1, two of them, VI and VII, differ by the position of the substituent in the thiophene ring [21]. A series of 4-amino-thieno [2,3-d]pyrimidines linked to N,N’diaryl urea moieties has been discovered. The performed cytotoxicity study showed that the compounds inhibit the vascular endothelial growth factor (VEGF)/kinase insert domain-containing receptor (KDR) and platelet-derived growth factor (PDGF) receptor. The IC_50_ of most active derivative IV (Figure 1) was 3 nM. It was estimated that many of the compounds demonstrate antitumor activity against the HT1080 human fibrosarcoma tumor growth model in vivo and have favorable pharmacokinetic (PK) profiles [24].

The development of multidrug resistance (MDR) is a serious hindrance to cancer treatment and leads to reduced sensitivity of cancer cells to the anticancer agents. The prevalent mechanism of MDR signifies an overexpression of ATP-binding cassette transporters (ABC). A panel of compounds that can reverse MDR mediated by ABC transporters has been published [25,26]. It was found that 4-amino-thienopyrimidine V (QB13) (Figure 1) acts as a modulator of ABC efflux pumps. The EC_50_ value of this compound in the daunorubicin accumulation assay was 1.02 μM. Through modification of the structure of QB13, a series of 4-amino-thieno [2,3-d]pyrimidines was designed, synthesized and evaluated as activators of P-gp mediated daunorubicin transport. Structural alteration in different positions of the scaffolds was well-tolerated by the transporters [26,27].

As the discussed PTKs are therapeutical targets in cancer treatment, and on the basis of the above-mentioned thienopyrimidines’ inhibitory properties, we set ourselves the objective to design and synthesize 4-amino-thieno [2,3-d]pyrimidines in order to study both their activity against human cancer cell lines and their effects on normal cell lines. This investigation is a continuation of our foregoing study of the cytotoxicity effects of thienopyrimidines on human cancer cell lines (MDA-MB-231, MCF-7, HT-29, HeLa, HepG2) as well as towards normal human cells [28,29].

In this paper we report the development of some new ethyl 4-amino-5-methyl-2-substituted-thieno [2,3-d]pyrimidine-6-carboxylates. The choice of these structures was due to the fact that thieno [2,3-d]pyrimidines substituted in the thiophene ring with a methyl and an ester group and an amino group at the fourth position of the pyrimidine cycle are less studied than tetrahydrobenzothienopyrimidin-4-one derivatives. Our hypothesis is that these structures could lead to compounds with good anticancer properties.

## 2. Results and Discussion

### 2.1. Chemistry

**Figure 2:**
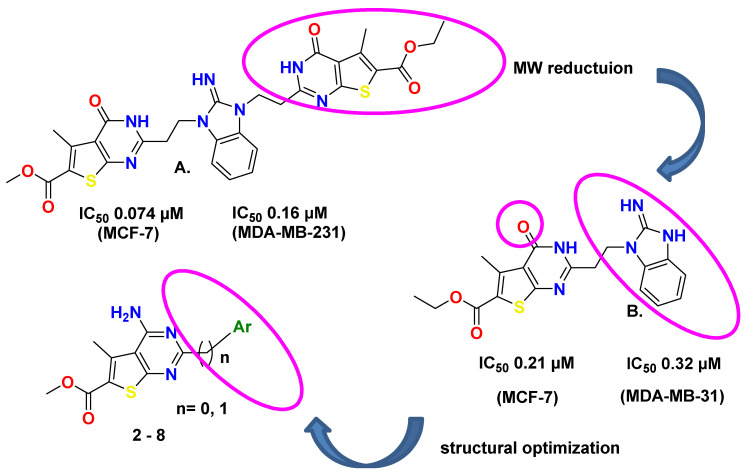
Structural optimization of compound **A** and **B**, MW (Molecular weight).

Based on the results published by us earlier, it was found that the best strategy to improve the antiproliferative potential of the already developed compounds is to convert bis-thienopyrimidine **A** into a monosubstituted compound. However, the compound **B** thus obtained did not show a better effect than **A**, so we undertook an optimization of the structure, as a result of which compounds **2**–**8** were generated (Figure 2).

**Scheme 1:**
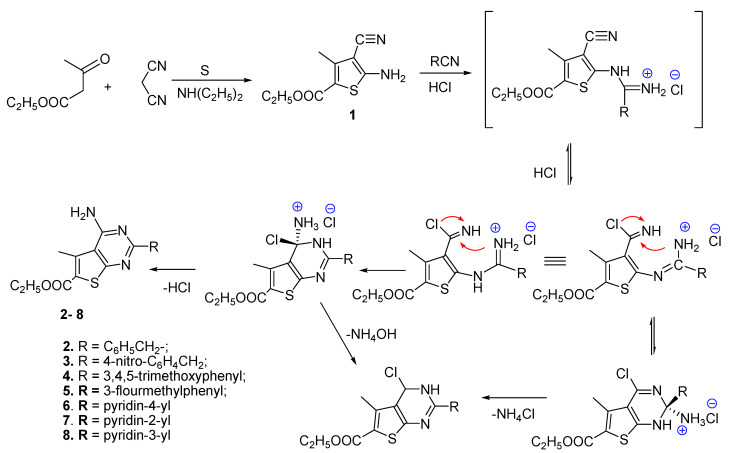
Synthesis of ethyl 4-amino-5-methyl-thieno [2,3-d]pyrimidine-6 carboxylates **2**–**8**.

The synthesis of the target compounds was performed as outlined in Scheme 1. The cyclization of 2-amino-3-cyano-thiophene with aryl or arylalkyl nitriles in the presence of dry hydrogen chloride gas yields 4-amino-thienopyrimidines as opposed to the cyclization of ethyl 2-amino-thiophene-3-carboxylates. 2-Aminothiophene-3-carbonitrile was obtained by means of Gewald reaction [30,31,32,33,34].

The synthesis was carried out with reagents ratio of the 2-amino-thiophene-3-carbonitrile to the respective nitrile 1:1, in dry dioxane as solvent. Dry hydrogen chloride gas was passed through the reaction solution [35]. As it can be seen in Scheme 1, the reaction mechanism differs from that of the preparation of 2-aminothiophene-3-carboxylate. In the case of thiophene nitriles, the synthesis proceeds through several intermediates, with the possibility of forming a by-product, namely 4-chloro-thienopyrimidine. This is the reason why lower yields were observed in this series in comparison to 4-oxo-thieno [2,3-d]pyrimidines [36]. The structure of the compounds was determined by IR, ^1^H NMR and ^13^C NMR spectra (see Supplementary Materials). The characteristic absorption for the ester carbonyl group of the synthesized compounds occurs in the range from 1725 cm^−1^ to 1692 cm^−1^. For the amino group, two vibration bands were observed, the first at 3400–3500 cm^−1^ and the second at 3390–3250 cm^−1^. The chemical shifts and the splitting patterns for the ester ethyl group are triplet for CH_3_ and quartet for CH_2_ group, respectively.

### 2.2. Molecular Structure Characterization

Characterization of the molecular structure of drug candidates, including tautomerism, can provide important insights in their possible interaction with the biological targets and might help in the tuning of a desired pharmacological activity. In the literature, it was already discussed that the precise structure of several drugs depends on the tautomeric equilibria affected by the medium polarity, and the ability of solvents to hydrogen-bond with each tautomer [37]. The consideration of the medium polarity by itself is very important, since a biological system may involve an aqueous medium (blood or plasma) or a nonprotic medium such as a cell membrane or enzymatic reaction center.

**Figure 3:**
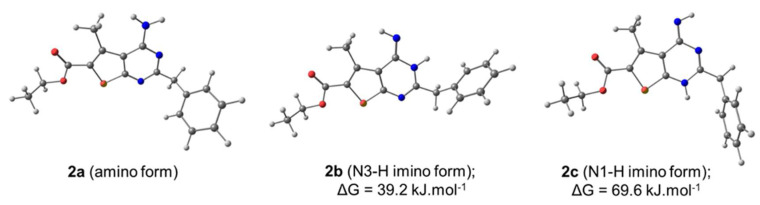
Tautomeric forms of compound **2**.

The most likely conformers of the molecules studied (including amino-imine tautomerism) were constructed and energy-minimized using Avogadro software [38]. Based on the energy analysis, the most stable conformers were selected and optimized by means of density functional theory (DFT) methods [39]. It is well established that the most aminohetero-aromatic compounds exist predominantly in amino form in aqueous solution or crystalline state [37,40]. In consent with this, in the IR spectra of the ethyl 4-amino-5-methyl-thieno [2,3-d]pyrimidine-6 carboxylates **2**–**8** two absorption bands were observed around 3400–3500 cm^−1^ and 3390–3250 cm^−1^. In order to estimate the tautomeric equilibrium in nonpolar phase, the amino form of compound **2** (**2a**) and two imino tautomers (**2b** and **2c**) were optimized in gas phase at B3LYP/6-311++G** level of theory (Figure 3).

The calculated relative free energies (ΔG) of the tautomeric forms of compound **2** suggested that the amino form is more favorable in a nonpolar environment as well, and due to the larger energy differences the imino forms are not expected to be present.

**Figure 4:**
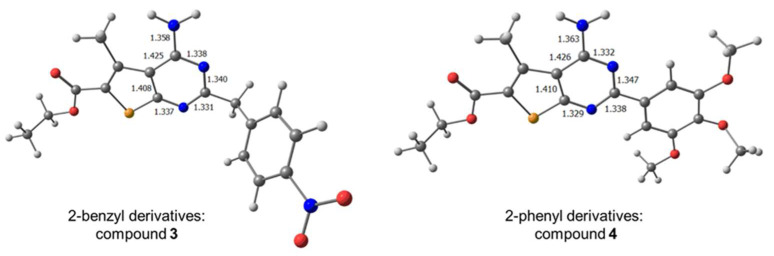
Bond-length alterations in the studied 4-amino-5-methyl-thieno [2,3-d]pyrimidine-6 carboxylates: 2-benzyl vs. 2-aryl substituted derivatives.

Based on the computations, it was also found that the conjugation with the phenyl ring, respectively to the pyridyl ring, slightly alters all bond lengths in the aminopyrimidine core in comparison to the benzyl derivatives (Figure 4), leading to elongation of the C-NH_2_ bond and the shortening of N3-C4 and N1-C6 bonds. The benzyl fragment is oriented almost perpendicularly to the plane of the thieno [2,3-d]pyrimidine fragment, while the aryl (phenyl and pyridyl) rings lay in the same plane, implying a more rigid structure of the respective molecules.

The computationally estimated structural parameters of the 4-amino-thienopyrimidine core of the new 4-amino-5-methyl-thieno [2,3-d]pyrimidine-6 carboxylates agree well with the reported crystallographic structure of QB13 [26]. The gained structural insights can be summarized that for both the benzyl- and the aryl-substituted 4-amino-5-methyl-thieno [2,3-d]pyrimidine-6 carboxylates, it is expected that they would interact with biological targets in their 4-amino tautomeric forms regardless of the medium polarity, but the benzyl derivatives have more flexible molecular structure and can possibly provide more efficient contacts with the receptors.

### 2.3. Biological Evaluation

#### 2.3.1. Cyto- and Phototoxicity

In this study we choose to elucidate the effect of the newly synthesized compounds on a panel of breast cancer cell lines; moreover, we tested their cytotoxicity and phototoxicity on the BALB 3T3/23 cell line.

**Figure 5:**
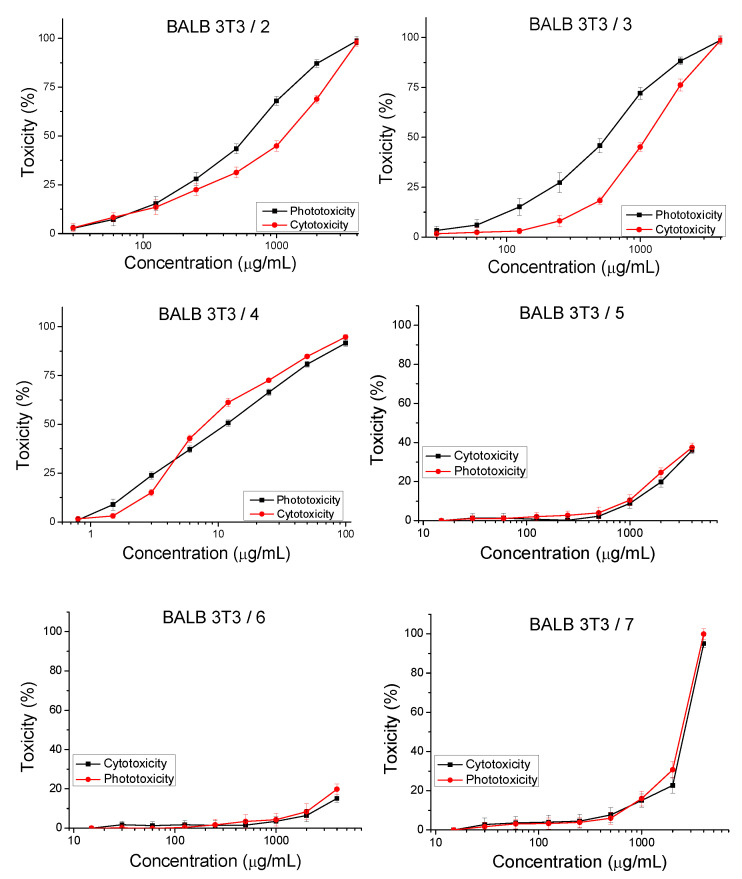
Cytotoxicity and phototoxicity of compound **2**–**7** to BALB 3T3 expressed in % relative to the negative control.

Some organic compounds exposed to sunlight can be converted into phototoxins as a result of light-induced changes in energy state or degradation of the molecule. The estimation of phototoxicity can serve as an indicator of the photostability of the studied thienopyrimidines when irradiated with UV light and as evidence that their effect on cells is only due to the cytotoxicity and not to phototoxicity. The compounds were studied for cytotoxicity by the 3T3 Neutral Red Reduction (NRU) phototoxicity test based on a comparison of the cytotoxicity of a chemical when tested in the presence and absence of noncytotoxic exposure to UVA/vis light. The cells were incubated with the test substances at a variable concentration: for compound **4** from 0.5 to 100 μg/mL, and for all the rest from 15 to 4000 μg/mL for 24 h. The cytotoxicity expressed in % relative to the negative control was determined and is given in Figure 5.

The results of the cytotoxicity and phototoxicity tests showed that the observed effects are of the dose-dependent type and are shown on Table 1.

The tested substances do not show phototoxicity, and most of them are not cytotoxic, which makes them suitable for subsequent experiments to establish antiproliferative and antitumor activity. As it can be seen, compounds **5** and **6** are identified as not cytotoxic versus BALB 3T3 cells.

#### 2.3.2. Antiproliferative Activity

In the next step of the study, we tested antiproliferative activity of the compounds 72 h after incubation of MCF-7 and MDA-MB-231 breast cancer cell lines and compared the results with those obtained by the use of MCF-10A (healthy human breast epithelial cells).

The compounds were studied for antiproliferative activity by standard MTT dye-reduction assay. Cell cultures from MCF-10A, MCF-7 and MDAMB-231 cell lines were incubated for 72 h in the presence of the tested substances in a wide range of concentrations. At the MCF-10A and MDA-MB-231 cell lines, compound **4** was applied in the range from 0.5 to 100 µg/mL, and all others from 4 to 1000 µg/mL.

**Figure 6:**
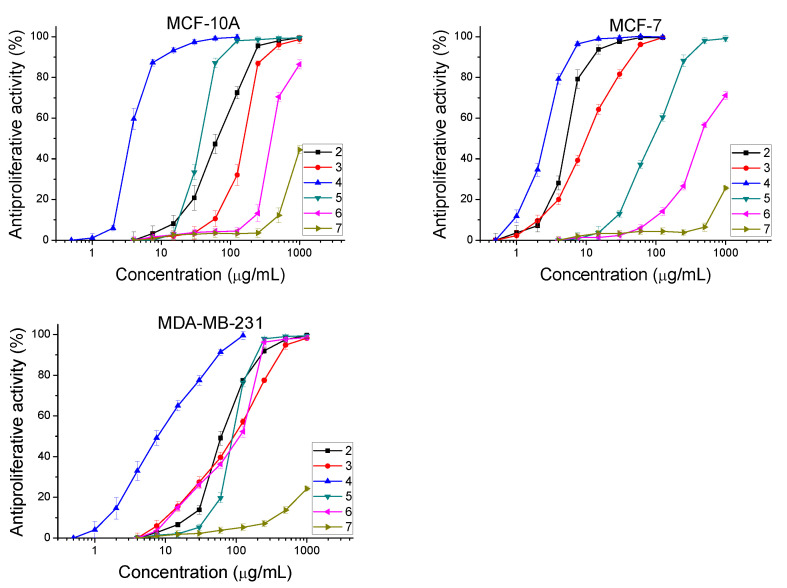
Antiproliferative activities of compounds **2**–**7** expressed in % relative to the negative control.

The MCF-7 cell line was treated with compounds **2**, **3** and **4** at concentrations from 0.5 to 100 µg/mL, and all others from 4 to 1000 µg/mL. The antiproliferative activity expressed in % relative to the negative control (untreated cells accepted as a 100% viable or 0% antiproliferative activity) was determined and shown on Figure 6.

The IC_50_ values of the mean were calculated and are presented in Table 2. In order to determine antitumor activity, as a reliable model and a control we used the MCF-10A cell line (normal human mammary epithelial cells).

As it can be seen in Table 1, compound **4**, containing three methoxy groups, is the most cytotoxic towards BALB 3T3 with IC_50_ value = 8.37 µg/mL, and in comparison with all tested compounds exhibited antiproliferative activity against other cell lines in the lowest concentrations. Therefore, the properties of this compound will not be discussed in detail. The thieno [2,3-d]pyrimidines **5** and **6** possessed the lowest cytotoxicity against BALB 3T3 with IC_50_ > 4000 µg/mL (>1 µM) and IC_50_ > 4000 µg/mL (>13 µM) accordingly, followed by compound 7 with IC_50_ = 2754 µg/mL (8.8 µM).

With regard to the MCF-7 cell line, the best antiproliferative effect (excluding compound **4**) revealed aminothienopyrimidines **2** and **3**, which is about 16-fold (compound **2**) and 20 times (compound **3**) higher in respect to their antiproliferative activity against MCF-10A.

If the data for the antiproliferative activity against MDA-MB-231 are taken in consideration, it should be pointed out that compound **2** manifested the highest antiproliferative inhibition with IC_50_ = 18.28 µg/mL (IC_50_ = 0.056 µM). The amines **3** and **5** showed similar values of the IC_50_ inhibitory activity: 0.25 µM and 0.26 µM, respectively.

The IC_50_ values, found by treatment of MCF-10A, are used to calculate the selective index (SI), which assesses the potential of a substance to be used as an antitumor agent. To calculate the SI, the following formula was used:

SI = IC_50_ of MCF-10A/IC_50_ of tumor cells (1)

Results are presented in Table 3.

The highest selective index with respect to MCF-7 is shown by the compounds **3** (SI = 19.3) and **2** (SI = 15.8). The widely used in clinical practice cytostatic Cisplatin (positive control) showed SI = 2.36. With respect to MDA-MB-231, thienopyrimidines **2** and **6** revealed the highest index (SI = 3.7 and 3.54, respectively). The calculated selective index in the positive control (Cisplatin) is SI = 25. The high levels of the selective index, as well as the strong antiproliferative effect of substances 2 and 3 require a detailed study of their mechanism of action.

#### 2.3.3. Ligand Efficiency

Ligand efficiency (LE) is a parameter used in recent years to account for the effects of physicochemical properties (MW, lipophilicity and other) of a ligand in optimizing and increasing the efficiency of hits or leading compounds. Ligand efficiency (LE) was first proposed for selecting favorable fragments through comparing the values of average binding energy per atom. LE corresponds to the Gibbs free energy of binding per heavy atom, and may be calculated by the following equation:

LE = pIC_50_ × 1.37/HA heavy atom count (kcal/mol) (2)

HA denotes the heavy atom count, which is the number of nonhydrogen atoms.

Determination of binding efficiency can be performed by simply dividing pIC_50_ by HA to obtain the so-called ligand efficiency index, which is a unitless quantity (LEI; Equation (3)). The HA can be replaced with MW, thus obtaining a binding efficiency index (BEI) [41,42,43,44]:

LEI = pIC_50_/HA (3)

BEI = pIC_50_/MW (4)

Ligand lipophilicity efficiency (LLE), also known as lipophilic efficacy (LipE), can be interpreted as the ability to transfer the ligand from 1-octanol to the ligand-binding site. In addition, some of the limitations of the 1-octanol/water-separation system become less important when working within structural series [45,46,47,48].

LLE = pIC_50_ − cLogP (5)

Lipophilicity is an important property that affects the progress of drug discovery and the ability to develop defined candidates. LE and LLE are considered acceptable when their values are greater than 0.3 and 3.0, respectively [47].

Various physicochemical properties such as molecular weight (MW), topological polar surface area (TPSA), sum of O and N H-bond acceptors (N_HA_), sum of OH and NH H-bond donors (N_HD_), lipophilicity (clogP), LE and LLE of compounds **A**, **2**–**7** were calculated and compared to the cytotoxic effects observed in the MCF-7 test (Table 4 and Table 5).

Briefly, compounds **2** and **3** revealed higher antiproliferative activity to MCF-7 versus thienopyrimidine **A**, whose LE, LEI and BEI values are lower than those of **2** and **3**. The fact that all the three compounds have approximately the same values with respect to LLE is due to their approximately equal partition coefficients. As opposed to **2** and **3**, the bis(thienopyrimidinyl)—benzimidazole **A** deviates by three parameters (MW, TPSA and N_HA_) from Lipinski’s rule.

As a result of the data obtained, it can be noted that the structural modification of the compound A leads to derivatives with higher antiproliferative potential. Although compound **3** has a higher selectivity index, compound **2** demonstrated higher ligand efficacy, ligand lipophilicity and lower TPSA values; therefore, it emerges as the leading compound in this study.

The highest antiproliferative activity in the MDA-MB-31 test manifested compound **2** (IC_50_ = 0.056µM), while the thienopyrimidine **B** possessed IC_50_ value = 0.32 µM; furthermore, the amine **2** demonstrated higher results of LE, LEI and BEI versus compound **B**. The LEL value for B is greater than that of **2**, but if TPSA parameters are taken in consideration it should be noted that TPSA value of compound 2 is much lower than that of compound **B**, which defines it as a promising anti-MDA-MB-231 agent.

## 3. Materials and Methods

For the synthesis of the target compounds, the following commercial reagents have been used without further purification: ethyl acetoacetate, phenylacetonitrile, formamide, chloropropionitrile (Merck, Rahway, NJ, USA); ethyl cyanoacetate, benzonitrile, 3,4,5-methoxybenzonitrile, 4-nitrophenylacetonitrile propionitrile, 3-(trifluoromethyl)benzonitrile, 4-nitrophenylcarbonitrile (Flucka); 2-cyanopyridine 4-cyanopyridine; malononitrile (Alpha Aesar, Haverhill, MA, USA). All inorganic substances and organic solvents used were pure for synthesis or analysis (Macron, Merck).

Melting points were determined as the phase transition from solid to liquid at atmospheric pressure, on a Boetius PHMK 5 microscope heating table, in Celsius degrees ± 1.0 °C. The purity of all the compounds obtained, as well as the retention coefficients (Rf), were estimated on silica gel plates F254 or Al_2_O_3_ 60 (Merck, 0.2 mm). IR spectra were recorded by use of KBr tablets on a Varian Scimitar 1000 spectrophotometer. All ^1^H-NMR spectra were taken on a Bruker Avance DRX 250 spectrometer (Bruker, Faelanden, Switzerland) or Bruker Avance AV 600 (Bruker, Faelanden, Switzerland). Chemical shifts are expressed to tetramethylsilane (TMS) and are represented in δ (ppm).

### 3.1. Synthesis

#### 3.1.1. General Procedure for the Synthesis of Ethyl 2-Amino-3-cyano-4-methylthiophene-5-carboxylate1

To a solution of 6.6 g (9.8 g, 0.1 mol) malonodinitrile and 12.7 mL (13.01 g, 0.1 mol) ethyl acetoacetate in 30 mL ethanol, 3.2 g sulfur was added. By cooling and vigorous stirring, an equimolar amount of the catalyst (diethylamine, 0.1 mol) was dripped for 30 min. The reaction mixture was stirred for further 75 min and the solution cooled, whereby the amino-thiophene crystallized. The precipitate was filtered off, washed thoroughly with water, recrystallized from ethanol and dried in vacuum. Yield: 53%; m.p. = 209–210 °C; Rf 0.67 (Benzene/MeOH = 5:1; IR (KBr, cm^−1^): 3401 (νNH), 3313 (νNH), 3202 (νNH), 2981 (νCH_3_), 2932 (νCH_2_), 2904 (νCH_3_), 2204 (νCN), 1674 (ν C=O), 1631 (νArH), 1546 (νArH), 1494 (νArH), 1416 (δCH_3_); 1313(C-N), 1272(C-N), 1191, 1107(ν C-O).

#### 3.1.2. General Procedure for the Synthesis of 2-Substituted 4-Amino Thieno [2,3-d]Pyrimidines: **2**–**7**

To 0.0094 mol of 2-amino-thiophene-3-carbonitrile dissolved in 20 mL dioxane, 0.0094 mol of the corresponding RCN derivative was added. Dry hydrochloric gas was passed through the reaction mixture for 6 h, by continuously stirring at room temperature. The solution was allowed to stand at room temperature for 12 h, then poured onto ice and neutralized with 10% (*v*/*v*) NH_4_OH to pH ~ 8. The precipitate formed was filtered off, washed extensively with water and dried in a vacuum dryer at 60 °C.

#### 3.1.3. Ethyl 4-Amino-2-benzyl-5-methyl-thieno[2,3-d]pyrimidine-6-carboxylate (**2**)

After filtration of the resulting precipitate, the filtrate was neutralized. During the neutralization, a resin formation around pH 5 was observed. The nonresinous portion was transferred to another vial and neutralized, and the resulting resinous mass was left in 25% (*v*/*v*) NH_4_OH solution for 12 h to crystallize. The resulting precipitates were recrystallized from methanol. Yield: 27%; m.p. = 159–161 °C; Rf 0.53 (EtOAc/n-Hexane = 1:1); IR (KBr, cm^−1^): 3503 (NH), 3296 (NH), 3122 (NH), 2983 (CH_3_), 2839 (CH_2_), 1711 cm^−1^ (C=O), 1641 (NH), 1557 (ArH), 1510 (ArH), 1251 (C–O), 734 (ArH); 1H ЯMP (CDCl_3_): δ = 1.42 (t, *J* = 7.1 Hz, 3H, CH_3_), 2.97 (s, 3H, CH_3_), 4.20 (s, 2H, CH_2_), 4.41 (q, *J* = 7.1 Hz, 2H, CH_2_), 6.96 (s, 2H, NH_2_), 7.26 (t, *J* = 7.4 Hz, ^1^H, ArH), 7.34 (t, *J* = 7.6 Hz, ^2^H, ArH), 7.47 (d, *J* = 7.4 Hz, 2H, ArH). Calculated for: C_17_H_17_N_3_O_2_S: C, 62.36; H, 5.23; N, 12.83; O, 9.77; S, 9.79; Found: C, 62.33; H, 5.26; N, 12.81; O, 9.78; S, 9.80.

#### 3.1.4. Ethyl 4-Amino-5-methyl-2-(4-nitrobenzyl) Thieno [2,3-d] Pyrimidine-6-carboxylate (**3**)

After neutralization, the resinous mass was left overnight in 25% (*v*/*v*) NH_4_OH, wherein the product crystallized. The pure product was obtained by recrystallization from dioxane. Yield: 27%; m.p. = 215–216 °C; Rf 0.48, EtOAC/n Hexane = 1:1); IR (KBr, cm^−1^): 3498 (NH), 3314 (NH), 3160 (NH), 2986 (CH_3_), 2935 (CH_2_), 1692 (C=O), 1644 (NH), 1552 (ArH), 1510 (ArH), 1347 (NO_2_), 1257; (C–O); ^1^H NMR (DMSO-d6, δ ppm): 1.29 (t, *J* = 7.1 Hz, 3H, CH_3_), 2.85 (s, 3H, CH_3_), 4.15 (s, 2H, CH_2_), 4.29 (q, *J* = 7.1 Hz, 2H, CH_2_), 7.56 (d, *J* = 8.7 Hz, 2H, ArH), 8.17 (d, *J* = 8.8 Hz, 2H, ArH); Elemental analysis: Calculated for C_17_H_16_N_4_O_4_S: C, 54.83; H, 4.33; N, 15.04; O, 17.19; S, 8.61; Found: C, 54.85; H, 4.30; N, 15.06; O, 17.20; S, 8.59.

#### 3.1.5. Ethyl 4-Amino-5-methyl-2-(3,4,5-trimethoxyphenyl)thieno [2,3-d]Pyrimidine-6-carboxylate (**4**)

Resin formation was observed upon neutralization; the resin was left in 25% (*v*/*v*) NH_4_OH for 12 h to crystallize. The product was purified by recrystallization from CH_3_COO, acetonotrile or benzene. Yields: 40%; m.p. = 191–193 °C; Rf 0.49(EtOAc/n-Hexane = 1:1); IR (KBr, cm^−1^): 3317 (NH), 3066 (ArH), 2989(CH_3_), 2945 (CH_2_), 2908 cm^−1^ (CH_3_), 2837 (CH_2_), 1718 cm^−1^ (C=O), 1653(NH), 1247 (C–O), 1131 (C–O); ^1^H NMR(DMSO-d6): δ = 1.31 (t, *J* = 7.1 Hz, 3H, CH_3_), 2.90 (s, *J* = 10.5 Hz, ^3^H, CH_3_), 3.72–3.89 (m, 9H, 3 OCH_3_), 4.31(q, *J* = 7.1 Hz, 2H, CH_2_), 7.39 (d, *J* = 1.2 Hz, 2H, ArH). Calculated for C_19_H_21_N_3_O_5_S; C, 56.56; H, 5.25; N, 10.42; O, 19.83; S, 7.95; Found: C, 56.57; H, 5.23; N, 10.40; O, 19.86; S, 7.92.

#### 3.1.6. Ethyl 4-Amino-5-methyl-2-(3-(trifluoromethyl) phenyl) Thieno [2,3-d] Pyrimidine-6-carboxylate (**5**)

The obtained resinous mass after the neutralization was left overnight in 25% (*v*/*v*) NH_4_OH, whereby the product crystallized. The pure compound was obtained by recrystallization from benzene or acetonitril. Yield: 28%; m.p. = 285–286 °C; Rf 0.43 (EtOAc/n-Hexane = 1:1); IR (KBr, cm ^−1^): 3498 (NH), 3433 (NH), 3315 (NH), 3168 (NH), 2986 (CH_3_), 2942 (CH_2_), 1721 cm^−1^ (C=O), 1691(NH), 1552 (ArH), 1509 (ArH), 1256 (C–O), 1122 (C−F); 1H NMR(DMSO-d6, δ ppm): 1.32 (t, *J* = 7.1 Hz, 3H, CH_2_), 2.86 (s, 3H CH_3_), 4.32 (q, *J* = 7.1 Hz, 2H, CH_2_), 7.80 (t, *J* = 7.9 Hz, 1H, ArH), 7.99 (d, *J* = 7.8 Hz, 1H, ArH), 8.44 (d, *J* = 7.9 Hz, 1H, ArH), 8.50 (s, 1H, ArH), 6.75 (s, 2H, NH_2_), 13.07 (s, 1H, NH, exchangeable with D_2_O). Calculated for: C_17_H_14_F_3_N_3_O_2_S: C, 53.54; H, 3.70; F, 14.94; N, 11.02; O, 8.39; S, 8.41; Found: C, 53.57; H, 3.72; F, 14.91; N, 11.05; O, 8.41; S, 8.39.

#### 3.1.7. Ethyl 4-Amino-5-methyl-2-(pyridin-4-yl)thieno[2,3-d]pyrimidine-6-carboxylate hydrochloride (**6**)

The thienopyrimidine crystallized in the course of reaction; the precipitate was filtered off and recrystallized from CH_3_COOH. Yield 39%; m.p. = 259–261 °C; Rf 0.67 (Benzene/MeOH = 5:1); IR (KBr, cm^−1^): 3472 (NH), 3263 (NH), 3191 (NH), 3068 (ArH), 2978 (CH_3_), 2884 (CH_2_), 2553 (=NH^+^), 1713 (C=O), 1631 (NH), 1556 (ArH), 1245 (C–O); 1H NMR (DMSO-d6, δ ppm): 1.32 (t, *J* = 7.1 Hz, 3H, CH_3_), 2.93 (s, 3H, CH_3_), 4.33 (q, *J* = 7.1 Hz, 2H, CH_2_), 8.63 (d, *J* = 6.4 Hz, 2H,ArH), 8.98 (d, *J* = 6.2 Hz, 2H, ArH). C_15_H_15_ClN_4_O_2_S: C, 51.35; H, 4.31; Cl, 10.11; N, 15.97; O, 9.12; S, 9.14; Found: C, 51.33; H, 4.34; Cl, 10.13; N, 15.96; O, 9.10; S, 9.12.

#### 3.1.8. Ethyl 4-Amino-5-methyl-2-(pyridin—2-yl) Thieno[2,3-d]pyrimidine-6-carboxylate (**7**)

The compound crystallized in the course of reaction. The precipitate was filtered off, washed with dioxane and recrystallized from DMF. The filtrate was neutralized and re-crystallized from DMF or dioxane. Yield 56%; m.p. = 262–264 °C; Rf 0.78 (Benzene/MeOH = 5:1); IR (KBr, cm^−1^): 3395 (NH), 3350 (NH), 3173 (NH), 310 (ArH), 2986 CH), 2905 (CH), 1700 (C=O), 1646 (NH), 1604 (ArH), 1242 (C–O); 1H NMR(DMSO-d6, δ ppm): 1.33 (t, *J* = 7.1 Hz, 3H, CH3), 2.94 (s, 3H, CH3), 4.35 (q, *J* = 7.1 Hz, 2H, CH2), 7.90–7.95 (m, 1H, ArH), 8.44 (t, *J* = 7.8 Hz, 1H, ArH), 8.61 (d, *J* = 8.0 Hz, 1H, ArH), 8.85 (d, *J* = 4.4 Hz, 1H, ArH). Calculated for C15H14N4O2S: C, 57.31; H, 4.49; N, 17.82; O, 10.18; S, 10.20; Found: C, 57.34; H, 4.52; N, 17.80; O, 10.15; S, 10.18.

#### 3.1.9. Ethyl 4-Amino-5-methyl-2-(pyridin-3-yl)thieno [2,3-d]Pyrimidine-6-carboxylate (**8**)

The compound crystallized in the course of reaction. The precipitate was filtered off, washed with dioxane and recrystallized from DMF. The filtrate is neutralized and recrystallized from DMF or dioxane. Yield 50%; m.p. = 241–242 °C; IR (KBr): 3386 cm^−1^ (NH), 3302 cm^−1^ (NH), 3205 cm^−1^ (NH), 2977 cm^−1^ (CH), 1725 cm^−1^ (COOEt), 1629 cm^−1^ (NH), 1240 cm^−1^ (C–O); 1H ЯMP (DMSOd6): δ = 1.32 (t, *J* = 7.1 Hz, 3H, CH3), 2.91 (s, 3H, CH3), 4.32 (q, *J* = 7.1 Hz, 2H, CH2), 7.54 (dd, *J* = 8.0, 4.7 Hz, 1H, ArH), 8.63 (dt, *J* = 8.0, 1.9 Hz, 1H, ArH), 8.69 (dd, *J* =4.7, 1.6 Hz, 1H, ArH), 9.48 (d, *J* = 1.7 Hz, 1H, ArH); Calculated for C_15_H_14_N4O_2_S: C, 57.31; H, 4.49; N, 17.82; O, 10.18; S, 10.20; Found: C, 57.40; H, 4.47; N, 17.85; O, 10.20; S, 10.16.

### 3.2. Light Source

The light source used was a light emitting diode (LED) matrix, an artificial solar light simulator Helios-iO, model LE-9ND55-H—5500K (SERIC Ltd., Tokyo, Japan).

### 3.3. Chemicals

Cell-culture reagents were Dulbecco’s modified Eagle’s medium (DMEM), fetal bovine serum (FBS) and antibiotics (penicillin and streptomycin); the disposable consumables were supplied by Orange Scientific, Braine-l’Alleud, Belgium.

### 3.4. Cell Lines

All breast cell lines (MCF-10A, MCF-7 and MDA-MB-231) were purchased from the American Type Culture Collection—ATCC (Manassas, VA, USA). The MCF-7 and MDA-MB-231 were cultivated in Dulbecco’s Modified Eagle’s Medium (DMEM) supplemented with 10% fetal bovine serum (FBS), 1% sodium pyruvate, 1% MEM nonessential amino acids (NEAA) without antibiotics. The nontumorigenic breast cell line (MCF-10A) was cultivated in DMEM medium (Sigma-Aldrich, St. Louis, MO, USA) supplemented with 10% FBS, 1% sodium pyruvate, 1% nonessential amino acids (NEAA), 20 ng/mL human epithelia growth factor (hEGF), 10 μg/mL insulin, and 0.5 μg/mL hydrocortisone without antibiotics. All cell lines were maintained in a humidified atmosphere with 5% CO_2_ at 37 °C.

### 3.5. Cytotoxicity and Phototoxicity Testing

BALB/3T3 cells were cultured in 75 cm^2^ tissue-culture flasks in DMEM, 10% FBS, 2 mM glutamine and antibiotics (penicillin 100 U/mL and streptomycin 100 µg/mL) at 37 °C, 5% CO_2_ and 90% relative humidity. Cytotoxicity/phototoxicity was assessed by validated BALB/3T3 clone A31 Neutral Red Uptake Assay (3T3 NRU test) [49,50]. Briefly, cells were plated in a 96-well microtiter plate at a density of 1 × 10^4^ cells/100 µL/well and were incubated for 24 h. A wide concentration range of the test compounds was applied. In phototoxicity tests, 96-well plates were irradiated with dose 2.4 J/cm^2^ and the cells were incubated for additional 24 h. After treatment with Neutral Red medium, washing and treatment with the Ethanol/Acetic acid solution, the absorption was measured on a TECAN microplate reader (TECAN, Grödig, Austria) at wavelength 540 nm.

Cytotoxicity/phototoxicity were expressed as CC_50_/PC_50_ values (concentrations required for 50% cytotoxicity/phototoxicity), calculated using nonlinear regression analysis (GraphPad Software, Prism 8.0.2 (263), San Diego, CA, USA). The CC_50_ values can be used to calculate the PIF (photoirritancy factor) for each test substance, according to the following formula:

PIF = Cytotoxicity (CC_50_)/Phototoxicity (PC_50_) (6)

The statistical analysis included application of one-way ANOVA followed by Bonferroni’s post hoc test. *p* < 0.05 was accepted as the lowest level of statistical significance. All results are presented as mean ± SD.

### 3.6. In Vitro Antiproliferative Activity

Using the standard MTT dye-reduction assay, described by Mosmann [51] the antiproliferative activity was tested. The method is based on the metabolism of the tetrazolium salt MTT to insoluble formazan. The formazan absorption was registered using a microplate reader at λ = 540 nm. The measured absorption is an indicator of cell viability and metabolic activity. Antiproliferative activities were expressed as IC_50_ values (concentrations required for 50% inhibition of cell growth), calculated using nonlinear regression analysis (GraphPad Software, San Diego, CA, USA).

The statistical analysis included application of one-way ANOVA followed by Bonferroni’s post hoc test. *p* < 0.05 was accepted as the lowest level of statistical significance. All results are presented as mean ± SD.

All experiments were performed in triplicate.

### 3.7. Molecular Calculations

#### Quantum Chemical Calculation

Theoretical calculations were performed using the Gaussian 09 software package [52]. The geometry and vibrational frequencies of the molecules studied were performed using a gradient analytical technique, without any symmetric constraints. The results were obtained using B3LYP (Becke three-parameter hybrid functional combined with Lee–Yang–Parr correlation functional) and 6-311++G** basis set. The optimized structures were further characterized by analytical calculations of harmonic vibration frequencies at the same level of theory.

## 4. Conclusions

2-substituted-4-amino-thieno [2,3-d]pyrimidines were synthesized in order to investigate their cytotoxicity and phototoxicity against BALB 3T3 as well as their antiproliferative activity towards MCF-10A, MCF-7 and MDA-MB-31. The photoirritancy factor showed that all tested compounds are not phototoxic and that most of them are also not cytotoxic to BALB 3T3. The selective index (SI), which is a sign for the potential of a substance to be used as an antitumor agent, was calculated by using the data obtained from the MCF-10A test. Compounds **2** and **3** possessed the highest SI in respect to the MCF-7 cells. The highest antiproliferative activity against MCF-7 cells revealed compound **2** and **3** with IC_50_ values 0.013 µM and 0.023 µM, respectively. The selectivity index of compound **2** demonstrating the best antiproliferative activity to the MDA-MB-31 cell line (IC_50_ = 0.056 µM) is lower compared to its SI against MCF-7 cells. The data of the performed cytotoxicity and antiproliferative test, as well as the calculated values of LE, LEI, BEI and LLE parameters, confirmed that the chosen strategy for structural optimization of the 4-aminothienopyrimidine derivatives is correct.

## Figures and Tables

**Table 1:** Cyto- and phototoxicity assay data.

Cell Line	Compounds	CC_50_ of Mean ± SD (µg/mL)		PIF *
		Cytotoxicity	Phototoxicity	
BALB 3T3	**2**	1212 ± 103.3	632.3 ± 35.6	1.9
	**3**	1155 ± 57.8	578 ± 58.7	2
	**4**	8.37 ± 0.6	11.85 ± 0.8	0.7
	**5**	-	-	-
	**6**	-	-	-
	**7**	2754 ± 64.3	2552 ± 75.1	1.08

* PIF (Photoirritancy factor), PIF < 2 not phototoxic, PIF ≥ 2 and < 5 probable phototoxicity, PIF ≥ 5 phototoxic.

**Table 2:** Anti-proliferative activity data. IC_50_ values in µg/mL and µM.

Compounds	IC_50_ ± SD (μg/mL) Resp. in (µM)		
	Antiproliferative Activity		
	MCF-10A	MCF-7	MDA-MB-231
**2**	67.77 ± 9.1 (0.21 µM)	4.3 ± 0.11 (0.013 µM)	18.28 ± 1.58 (0.056 µM)
**3**	165.4 ± 8.81 (0.44 µM)	8.58 ± 0.55 (0.023 µM)	98.25 ± 4.73 (0.26 µM)
**4**	2.74 ± 0.13 (0.0068 µM)	2.01 ± 0.07 (0.005 µM)	6.43 ± 0.95 (0.016 µM)
**5**	39.23 ± 1.8 (0.1 µM)	95.94 ± 3.49 (0.25 µM)	94.94 ± 1.48 0.25 µM)
**6**	410.9 ± 5.1 (1.31 µM)	444.5 ± 9.2 (1.41 µM)	**116 ± 9.63** (0.37 µM)
**7**	>1000 -	>1000 -	>1000 -

**Table 3:** Determination of selective index (SI).

Compounds	Selective Index	
	MCF-7	MDA-MB-231
**2**	**15.8**	**3.7**
**3**	**19.3**	1.7
**4**	1.4	0.4
**5**	0.41	0.41
**6**	0.92	**3.54**
**7**	-	-

**Table 4:** Physicochemical properties and revealed LE and LLE of compounds **A**, **2**–**7** in MCF-7 test.

No	IC_50_ (µM)/ (pCI_50_)	LE kcal/mol	LEI	BEI	LLE	clogP	MW	TPSA	N_HA_	N_HD_
**2**	0.013 (7.88)	0.476	0.347	0.024	3.984	3.986	327.41	78.11	5	2
**3**	0.023 (7.64)	0.421	0.306	0.021	3.910	3.73	372.41	123.94	8	2
**4**	0.005 (8.30)	0.425	0.321	0.022	4.83	3.469	403.46	105.81	8	2
**5**	0.25 (6.60)	0.263	0.269	0.018	1.89	4.71	381.38	78.11	5	2
**6**	1.41 (5.85)	0.372	0.27	0.019	3.247	2.603	314.37	91.00	6	2
**A**	0.074 (7.13)	0.243	0.178	0.012	3.143	3.987	646.15	177.84	13	3

**Table 5:** LE and LLE of compounds **B**, **2**–**6** in MDA-MB-31 test.

No	IC_50_ (µM)	(pCI_50_)	LE kcal/mol	LEI	BEI	LLE	Vol
**2**	0.056	7.25	0.476	0.346	0.024	4.014	287.82
**3**	0.26	6.585	0.369	0.269	0.019	3.27	311.16
**4**	0.016	7.796	0.391	0.286	0.002	4.531	347.66
**5**	0.25	6.60	0.384	0.280	0.018	4.71	302.32
**6**	0.37	6.431	0.436	0.318	0.022	4.37	266.87
B *	0.32	6.444	0.342	0.28	0.018	5.64	344.08

B *: clogP = 1.36; TPSA—112.01; MW—399.48; N_HA_—8; N_HD_—4.

## Data Availability

Not applicable.

## Supplementary Materials

The following supporting information can be downloaded at: https://www.mdpi.com/article/10.3390/molecules27103314/s1, IR spectra (Figures S1a,b–S8a,b); ^1^HNMR spectra (Figures S9a,b–S15a,b).

## Sample Availability

All samples of the compound are not available from the authors.
