# Supplementary material for: Design, Cytotoxicity and Antiproliferative Activity of 4-Amino-5-methyl-thieno[2,3-d]pyrimidine-6-carboxylates against MFC-7 and MDA-MB-231 Breast Cancer Cell Lines
Source: Molecules. 2022 May 21;27(10):3314. doi: 10.3390/molecules27103314 (PMC9148072; doi:10.3390/molecules27103314)
Supplement: Supplementary file 1 [file molecules-27-03314-s001.zip › molecules-1705387-supplementary.pdf]

Design, Cytotoxicity and Antiproliferative Activity of 4-Amino-5-methyl-thieno[2,3-d]pyrimidine-6-carboxylates against MFC-7 and MDA-MB-231 Breast Cancer Cell Lines

Anelia Mavrova [1], Stephan Dimov [1], Inna Sulikovska [2], Denitsa Yancheva [3], Ivan Iliev [2,4], Iana Tsoneva [4], Galya Staneva [4] and Biliana Nikolova [4,*]

[1] Department of Organic Synthesis, University of Chemical Technology and Metallurgy, 8 Kliment Ohridski Blvd., 1756 Sofia, Bulgaria; anmav@abv.bg (A.M.); s_t_e_v_e@abv.bg (S.D.)

[2] Institute of Experimental Morphology, Pathology and Anthropology with Museum, Bulgarian Academy of Sciences, Acad. G. Bonchev Str., bl.25, 1113 Sofia, Bulgaria; inna_sulikovska@ukr.net (I.S.); taparsky@abv.bg (I.I.)

[3] Institute of Organic Chemistry with Centre of Phytochemistry, Bulgarian Academy of Sciences, Acad. G. Bonchev Str., bl. 9, 1113 Sofia, Bulgaria; denitsa.pantaleeva@orgchm.bas.bg

[4] Institute of Biophysics and Biomedical Engineering, Bulgarian Academy of Sciences, Acad. G. Bonchev Str., bl.21, 1113 Sofia, Bulgaria; itsoneva@bio21.bas.bg (I.T.); g_staneva@yahoo.com (G.S.)

* Correspondence: nikolova@bio21.bas.bg

Supplementary materials

Contents:

IR spectra (Figure S1-a,b – S8-a,b)……………………………………p.2-p.9

^{1}HNMR spectra (Figure S9-a,b – S15-a,b)……………………………..p.10-p.18

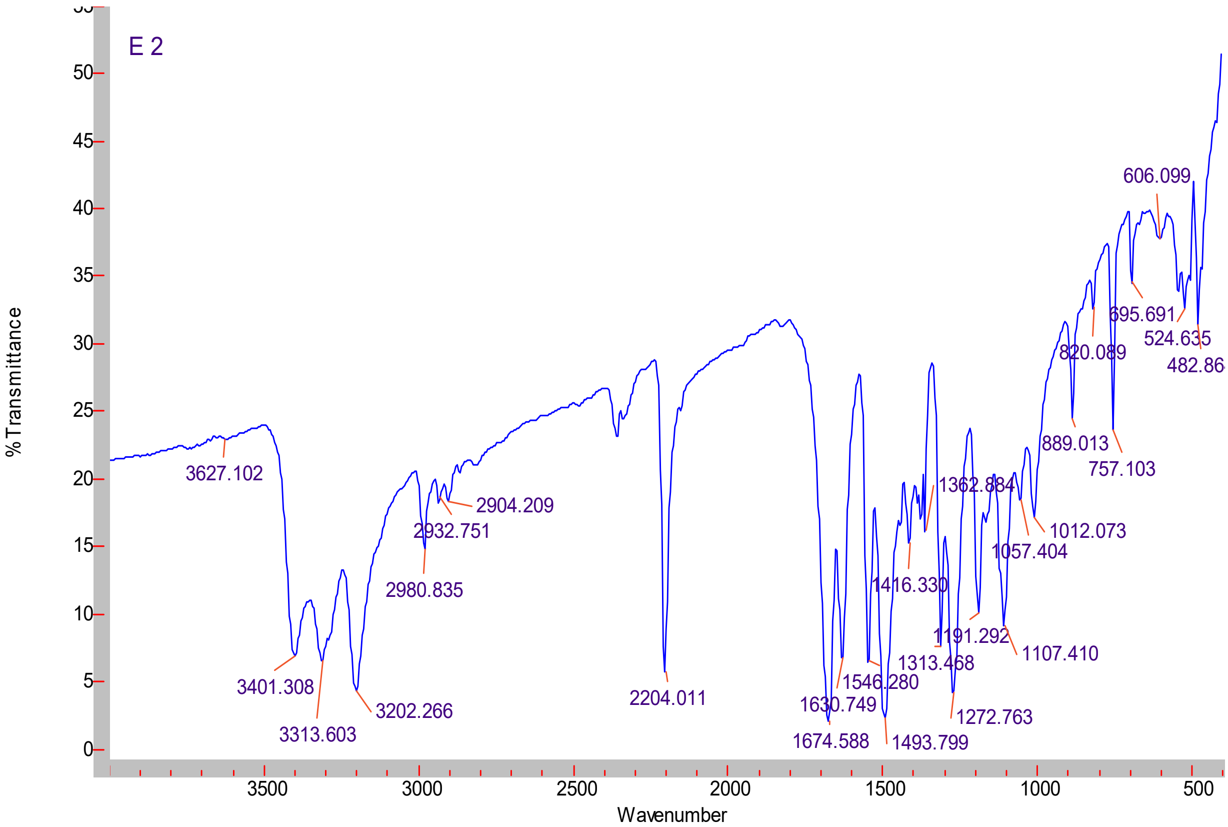

Figure S1-a IR spectrum of compounds **1**

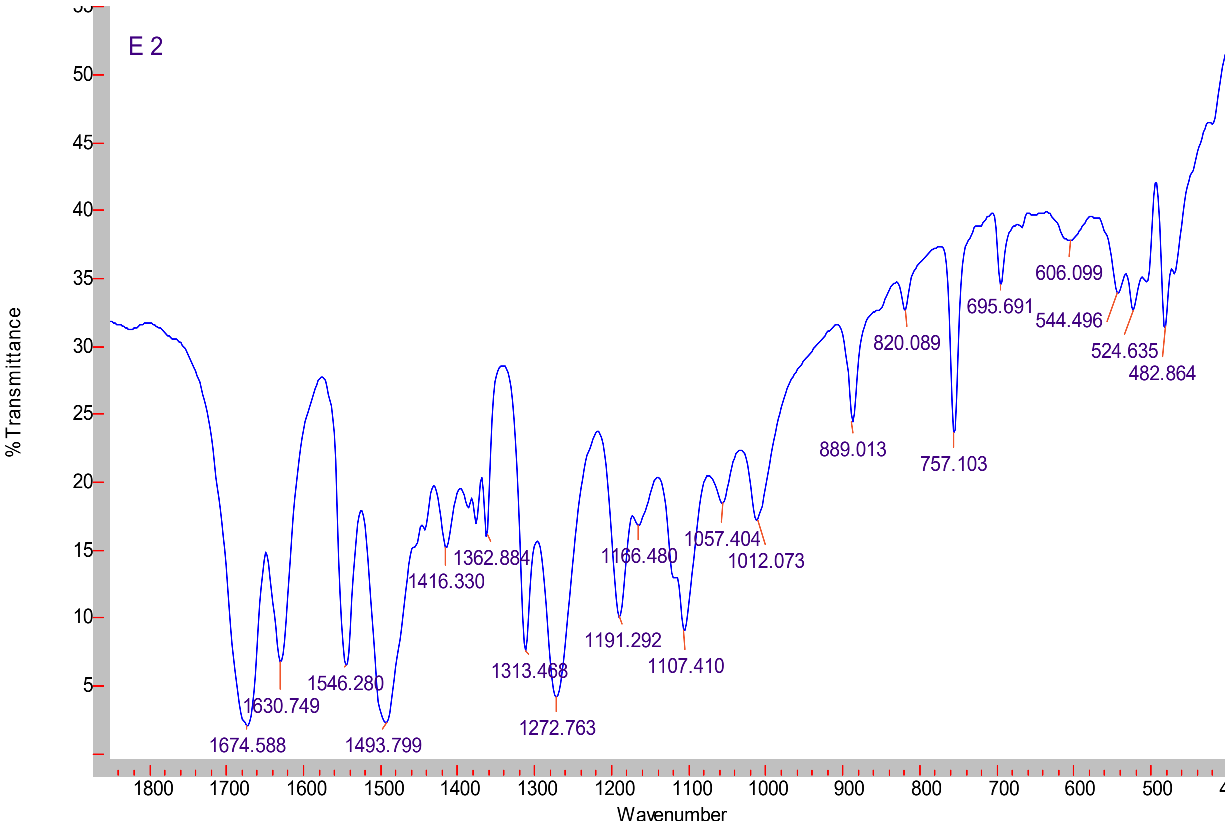

Figure S1-b IR spectrum of compounds **1**

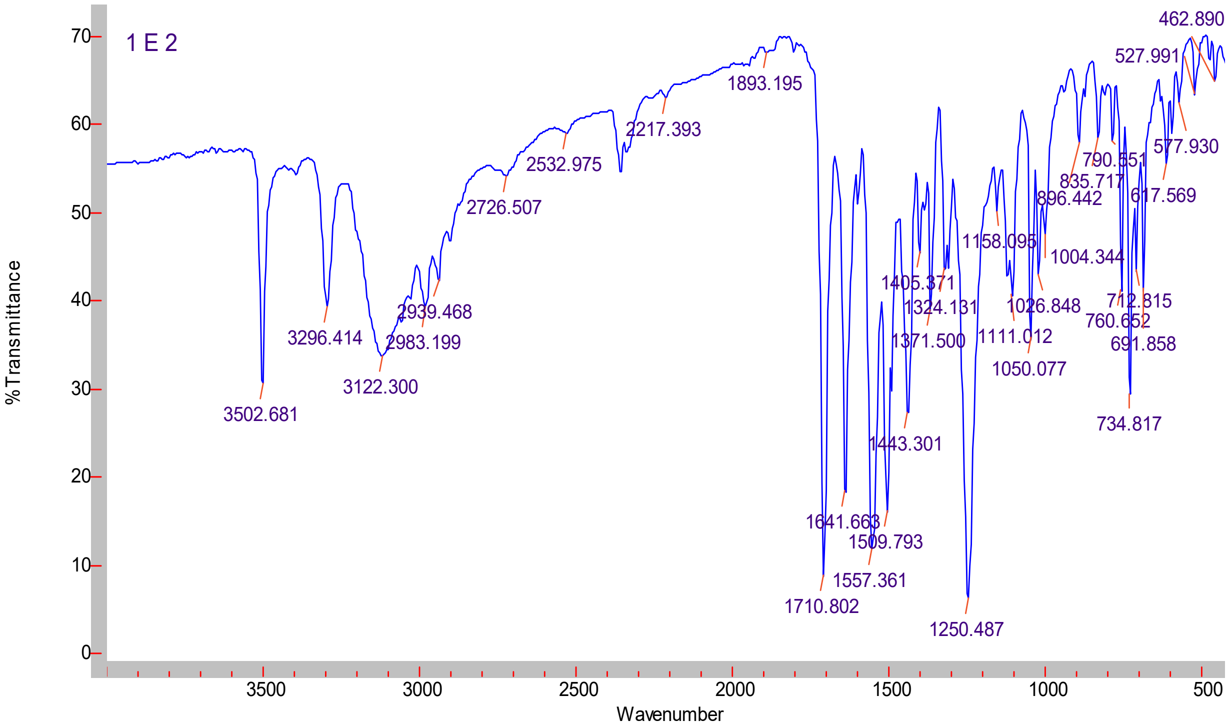

Fig S2-a IR spectrum of compound **2**

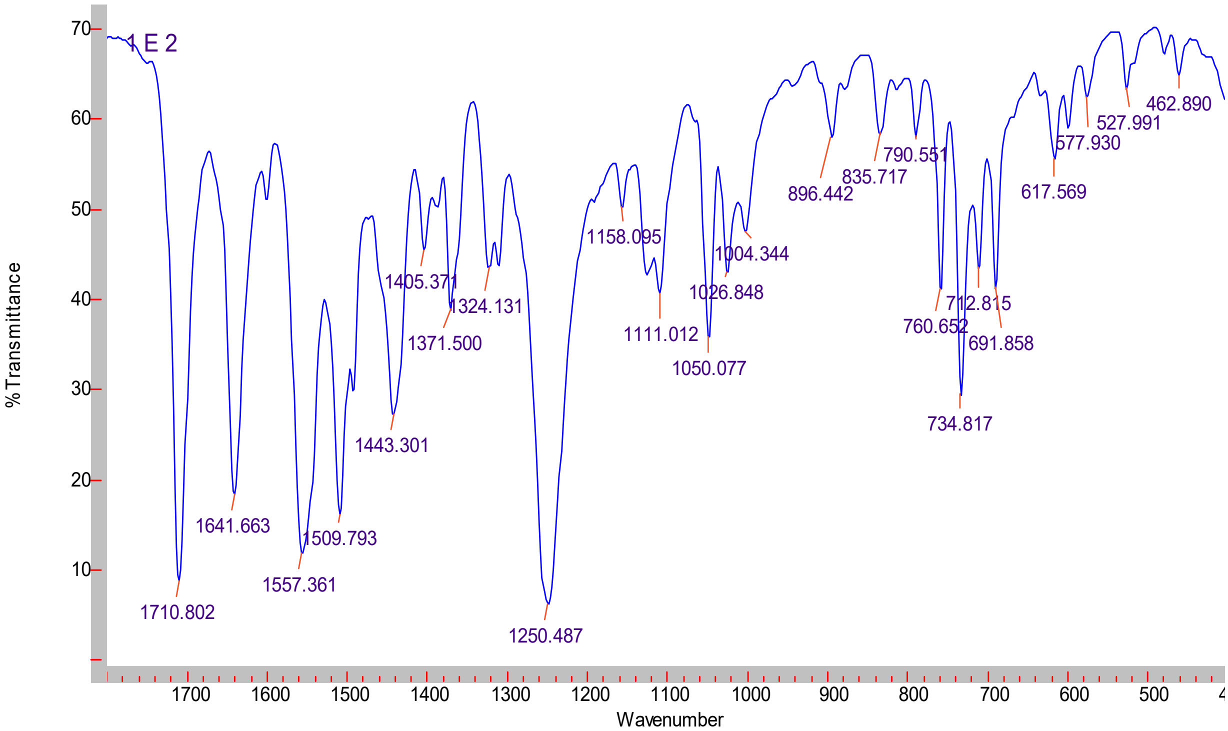

Figure S2-b IR spectrum of compound **2**

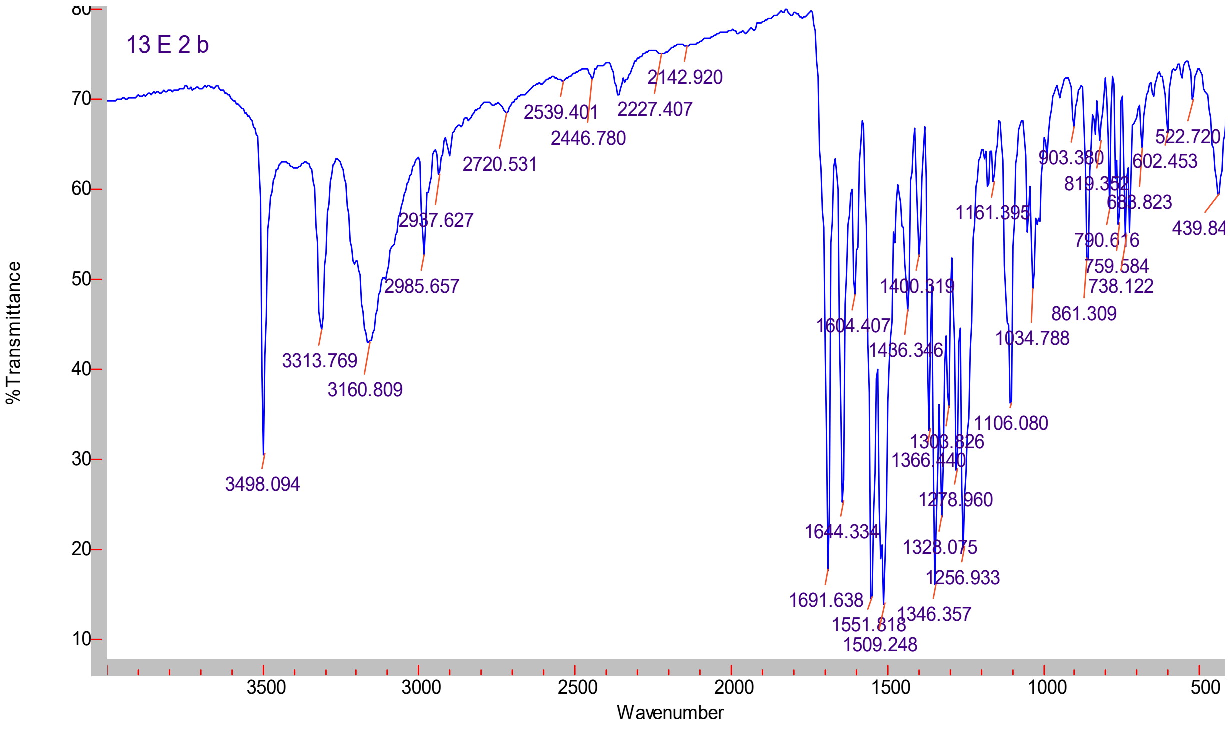

Figure S3-a IR spectrum of compound of compound **3**

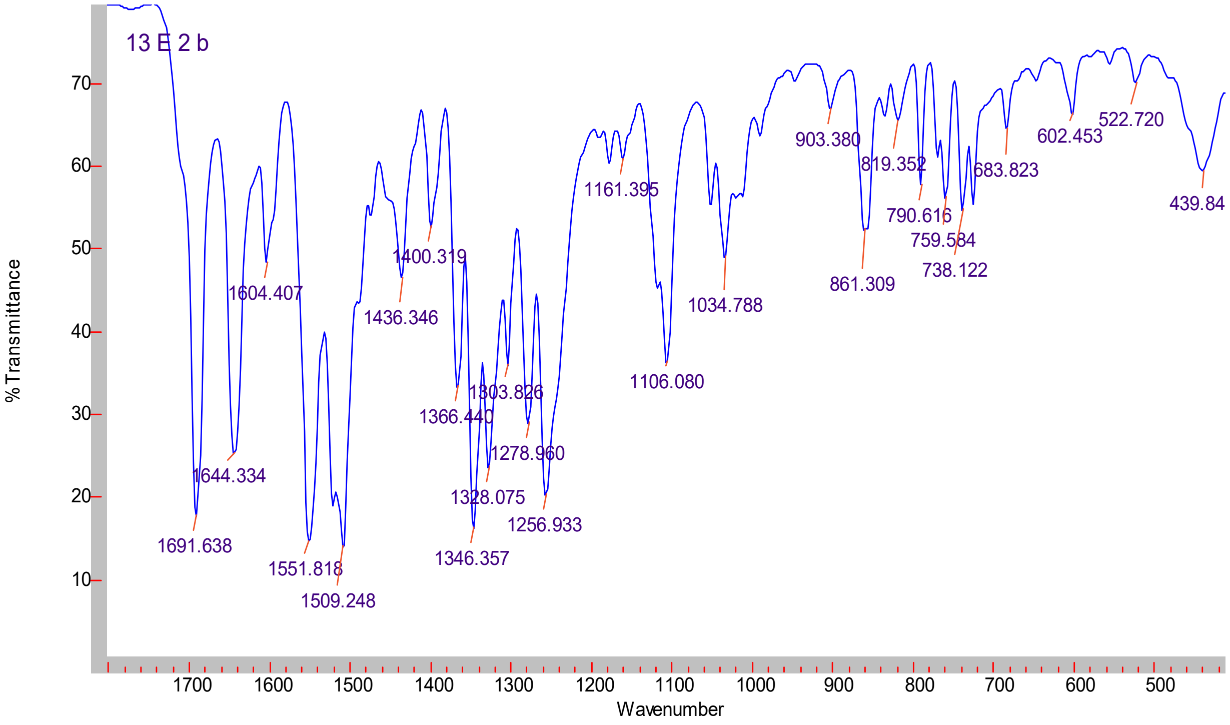

Figure S3-b IR spectrum of compound of compound **3**

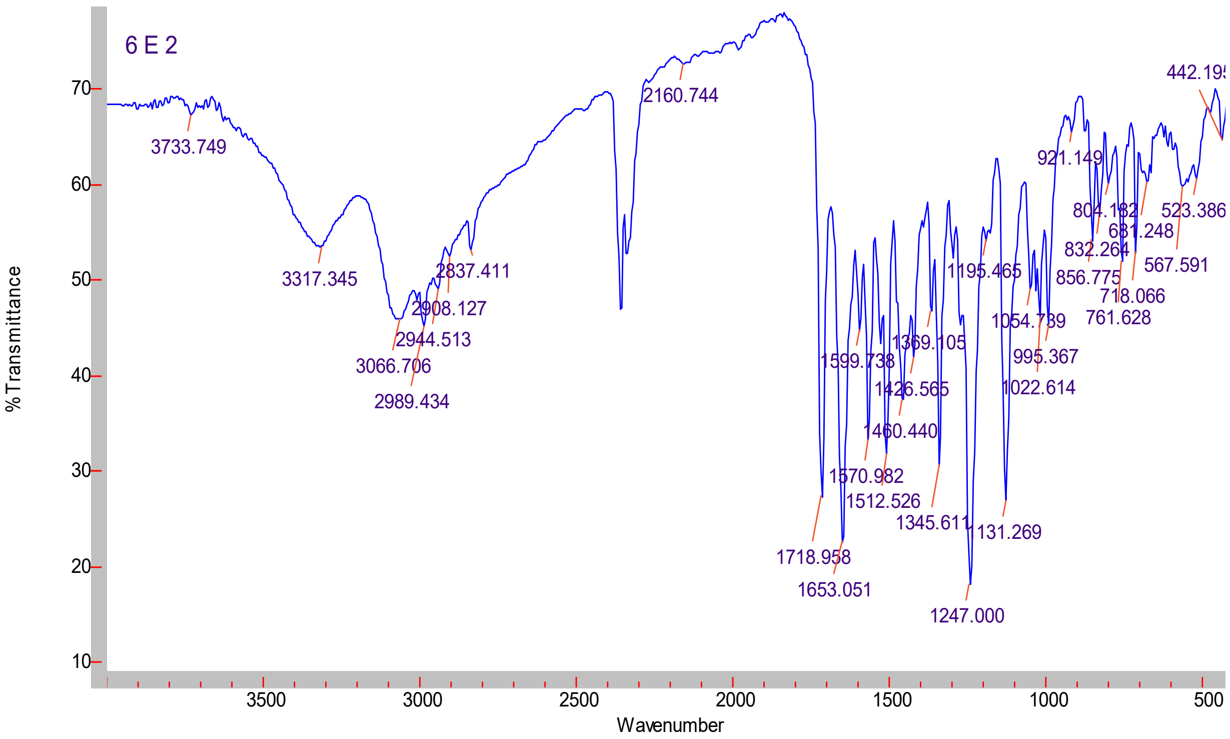

Figure S4-a IR spectrum of compound **4**

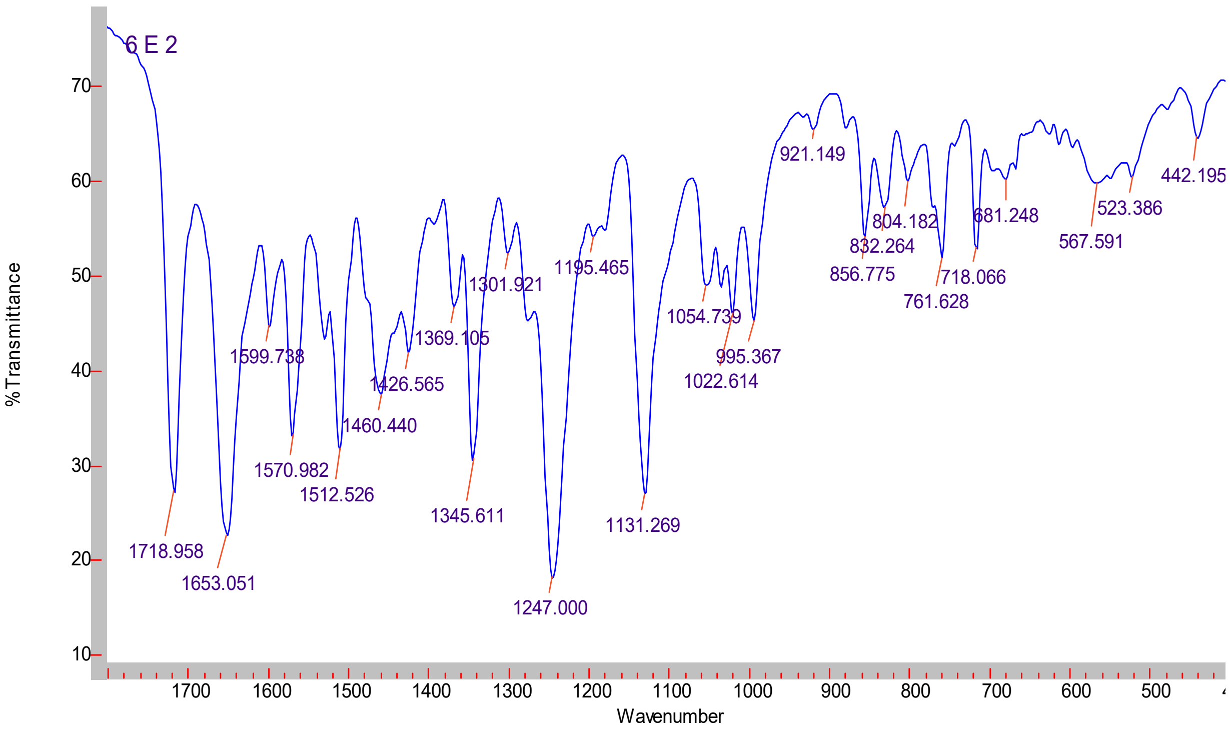

Figure S4-b IR spectrum of compound **4**

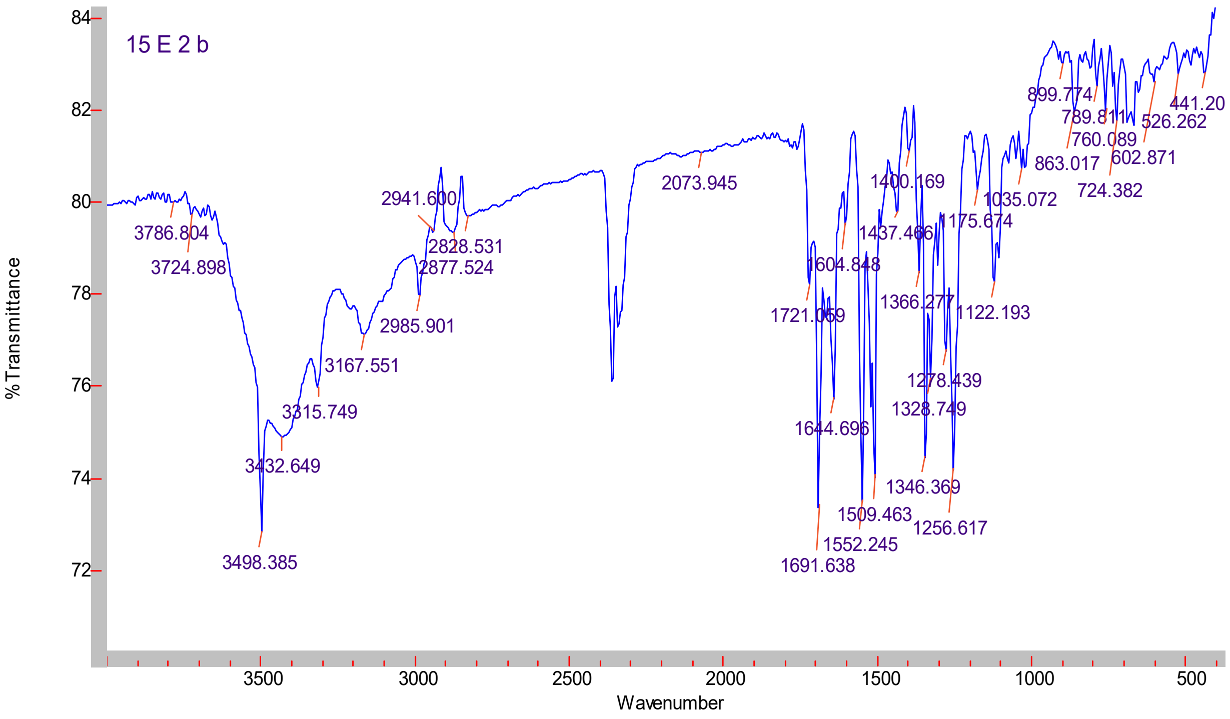

Figure S5-a IR spectrum of compound **5**

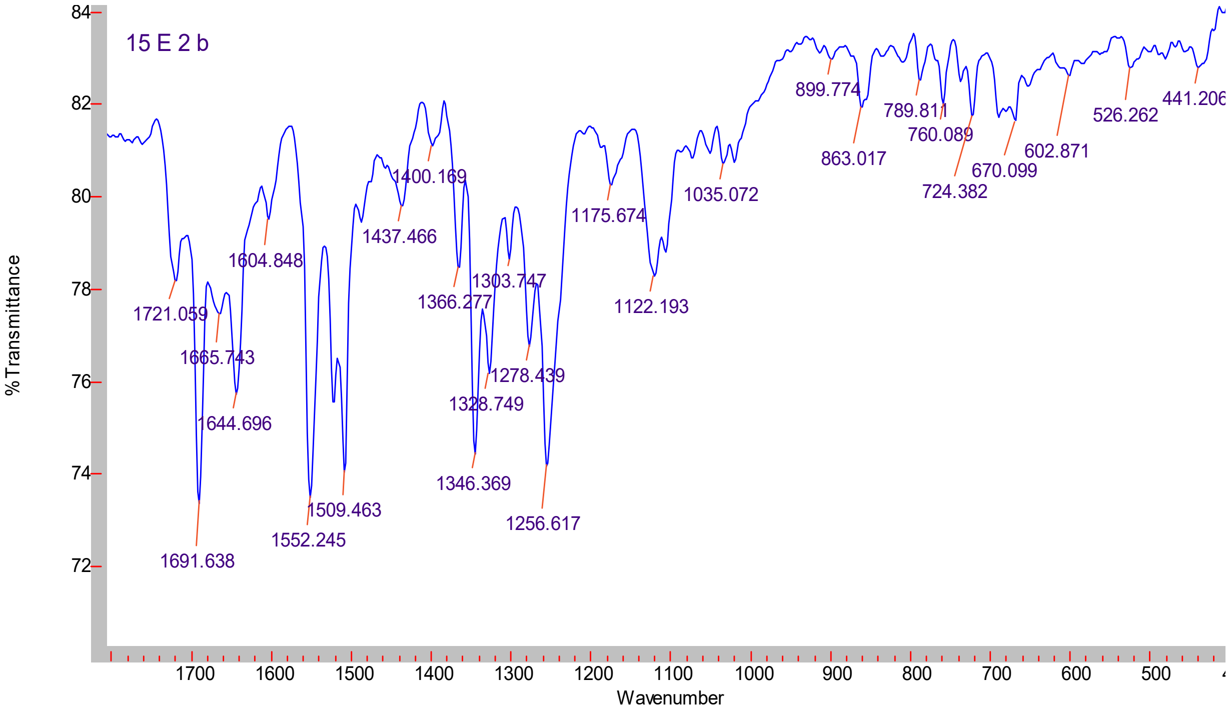

Figure S5-b IR spectrum of compound **5**

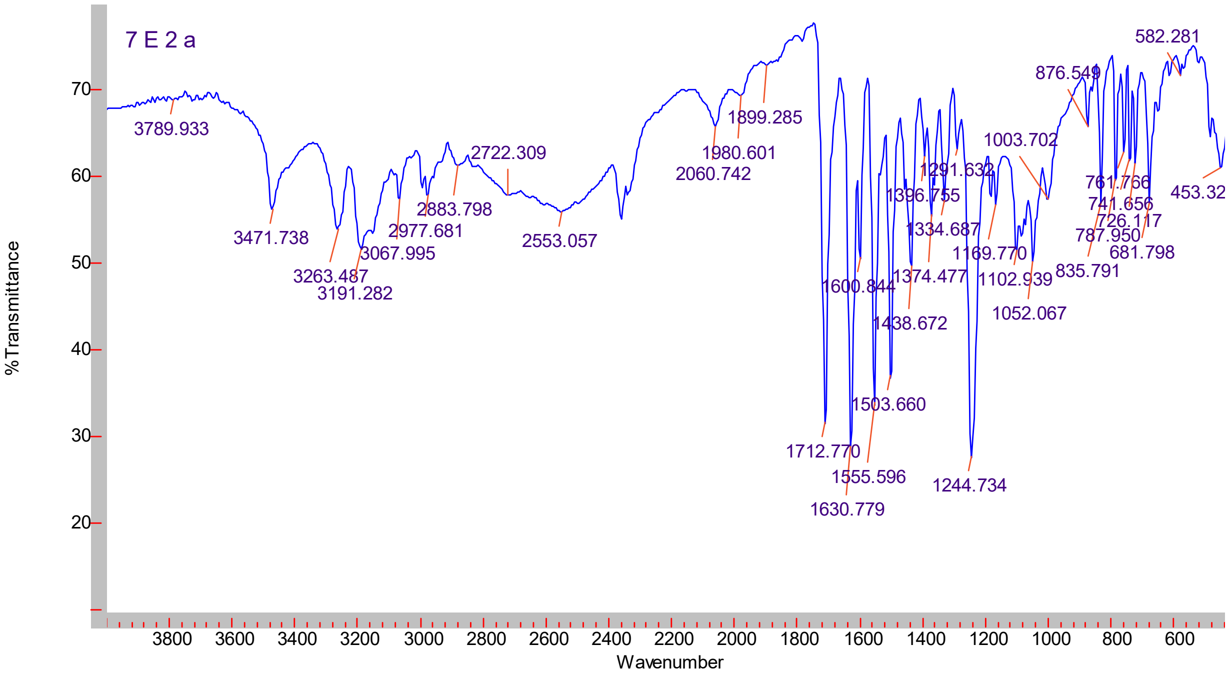

Figure S6-a IR spectrum of compound **6**

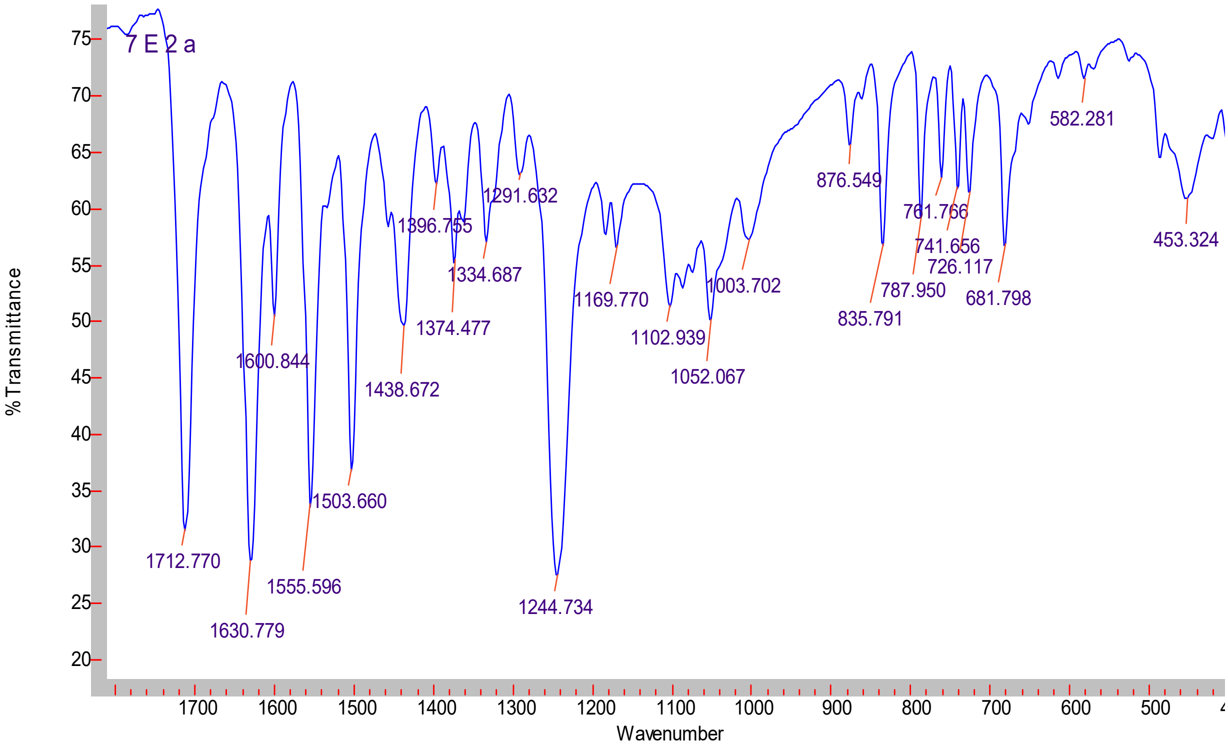

Figure S6-b IR spectrum of compound **6**

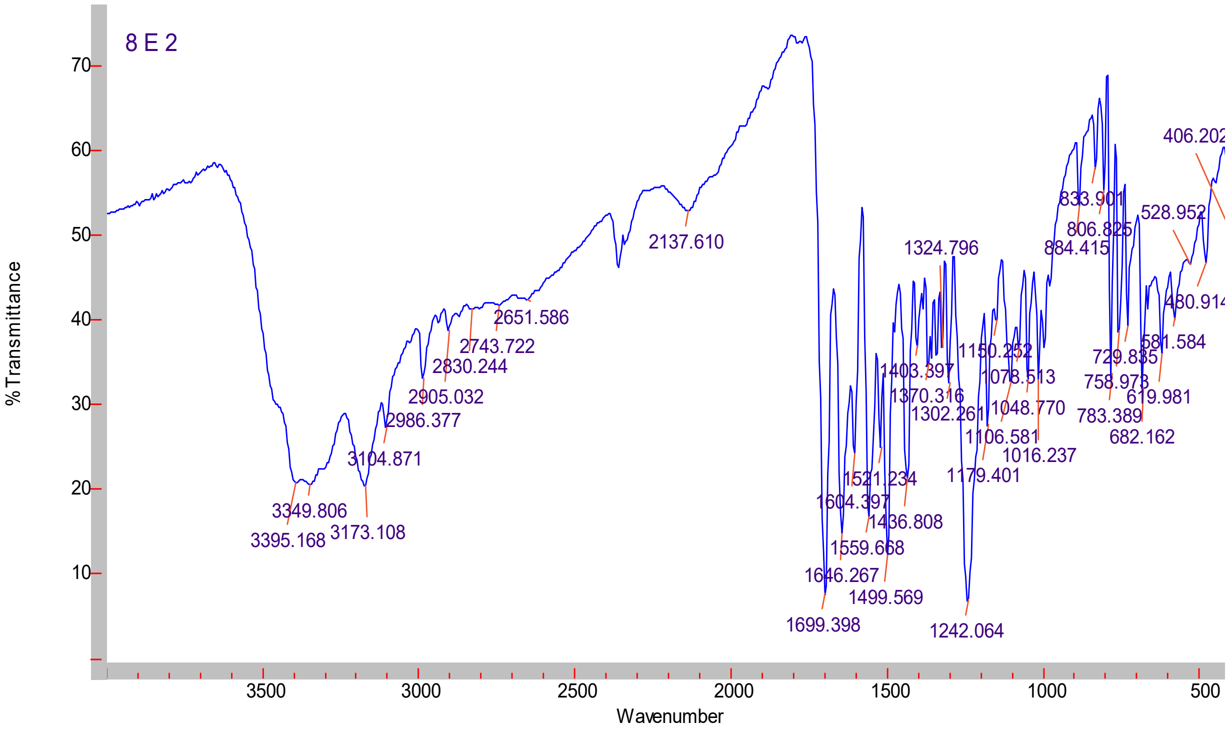

Figure S7-a IR spectrum of compound **7**

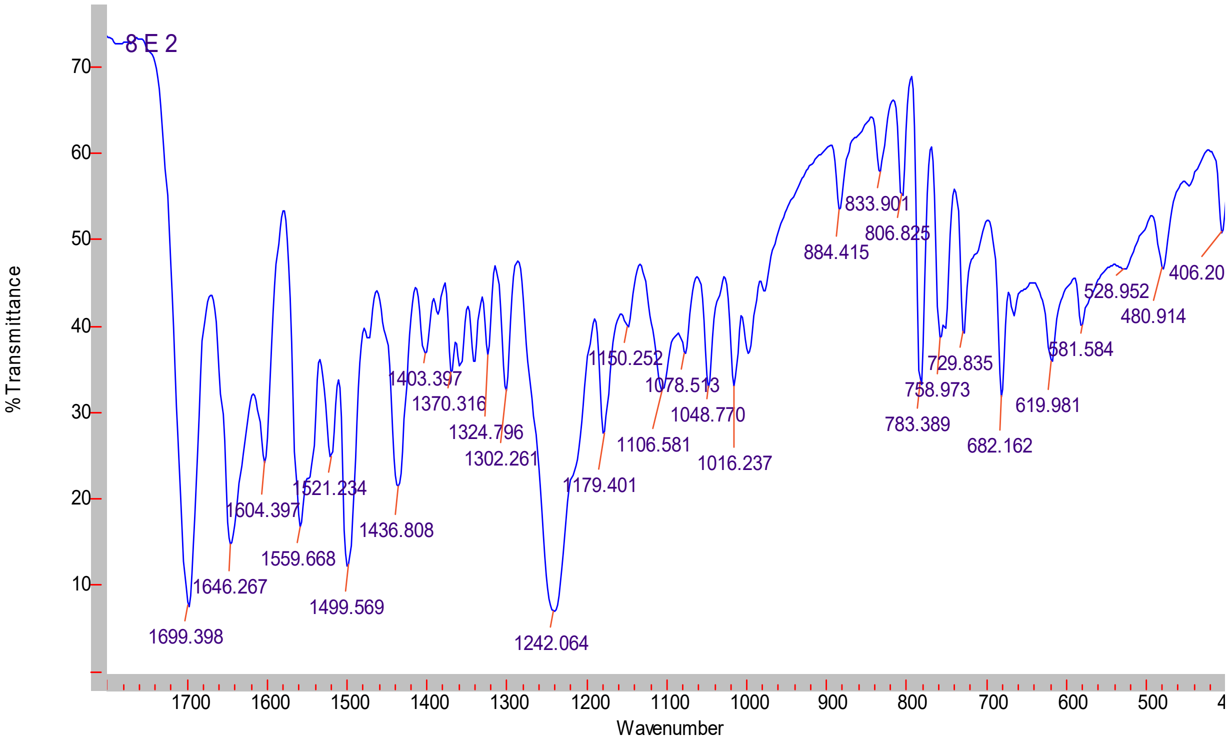

Figure S7-b IR spectrum of compound **7**

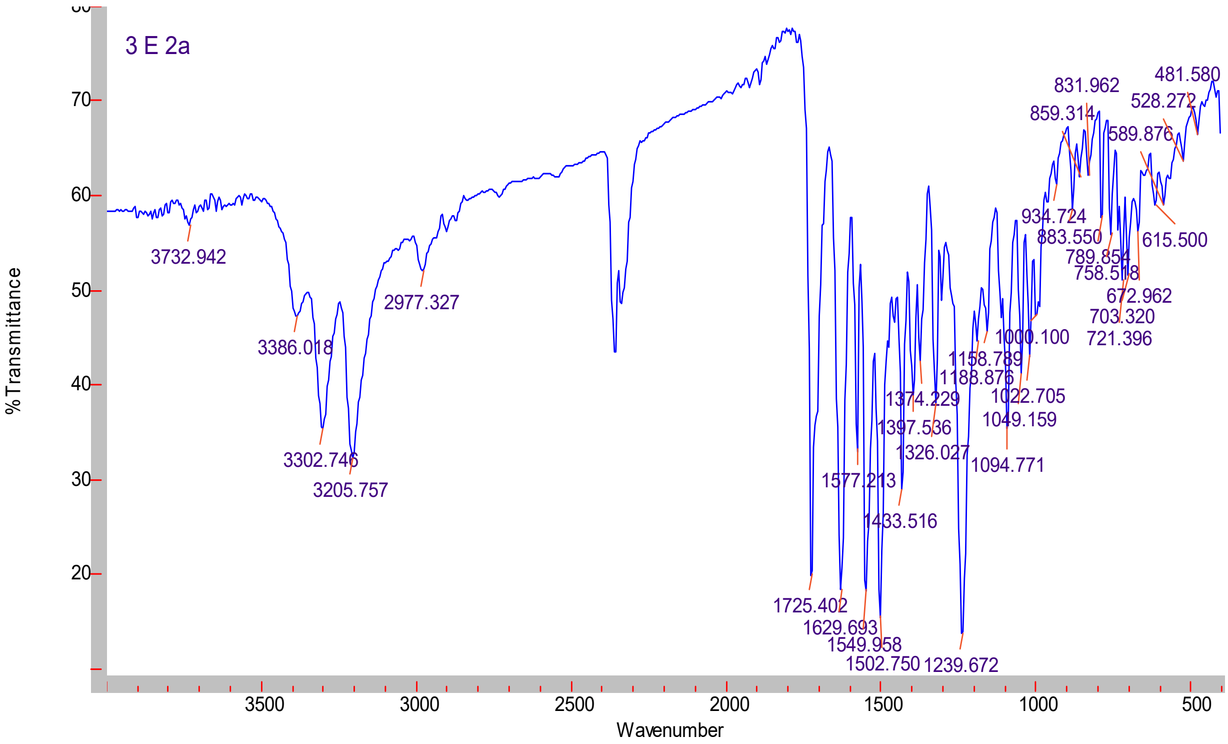

Figure S8-a IR spectrum of compound **8**

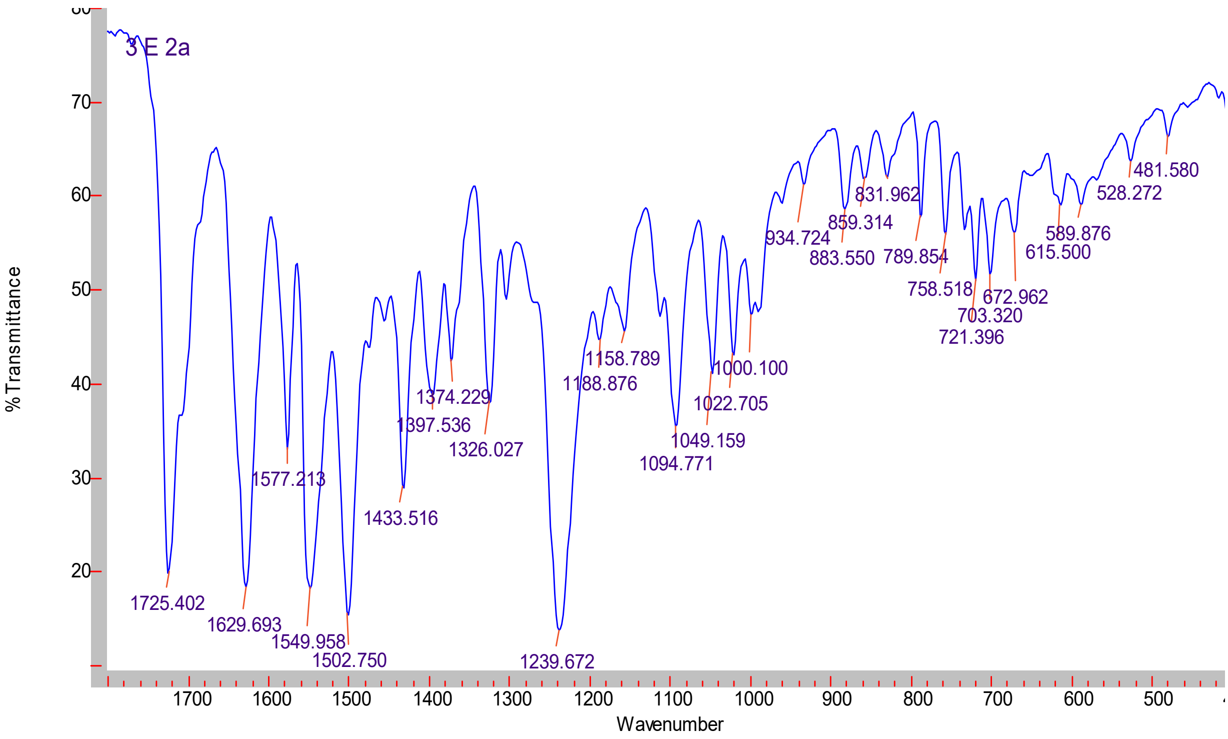

Figure S8-a IR spectrum of compound **8**

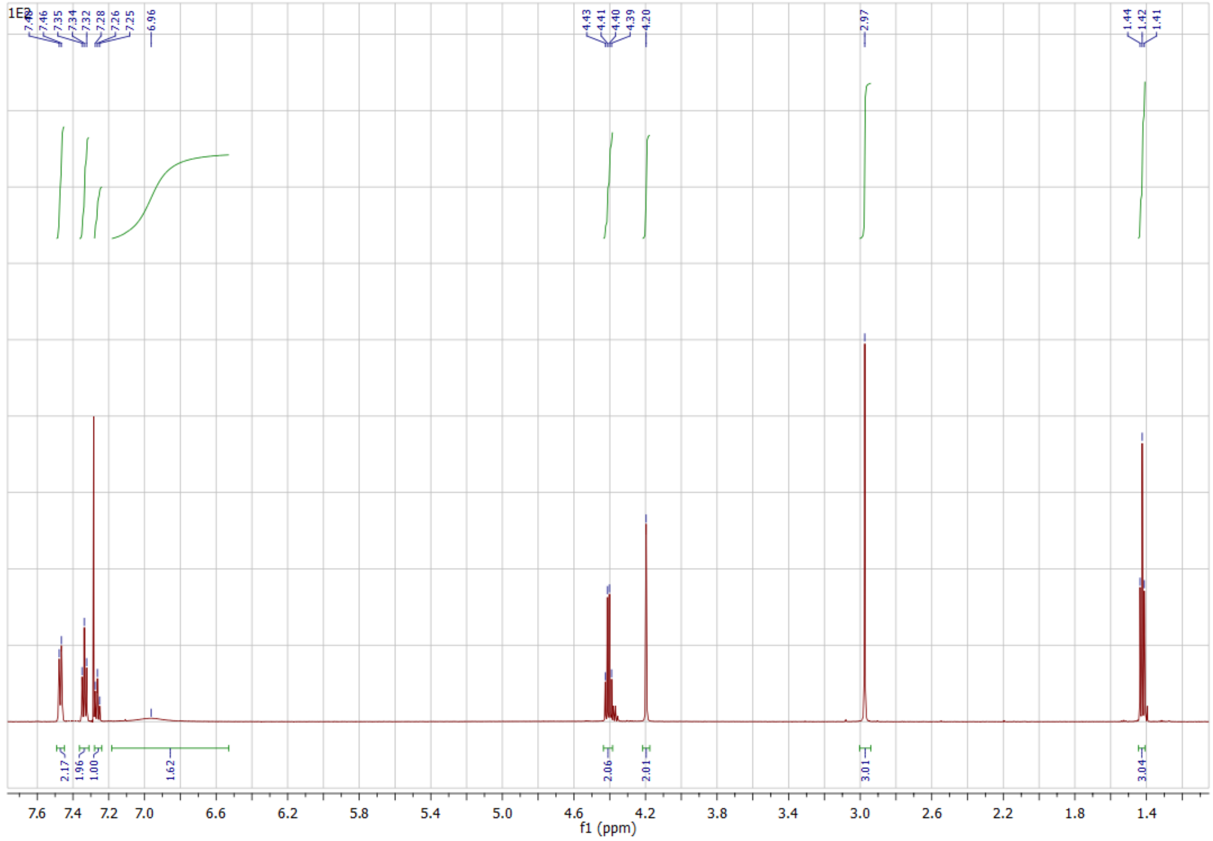

Figure S9-a ^{1}HNMR spectrum of compound **2**

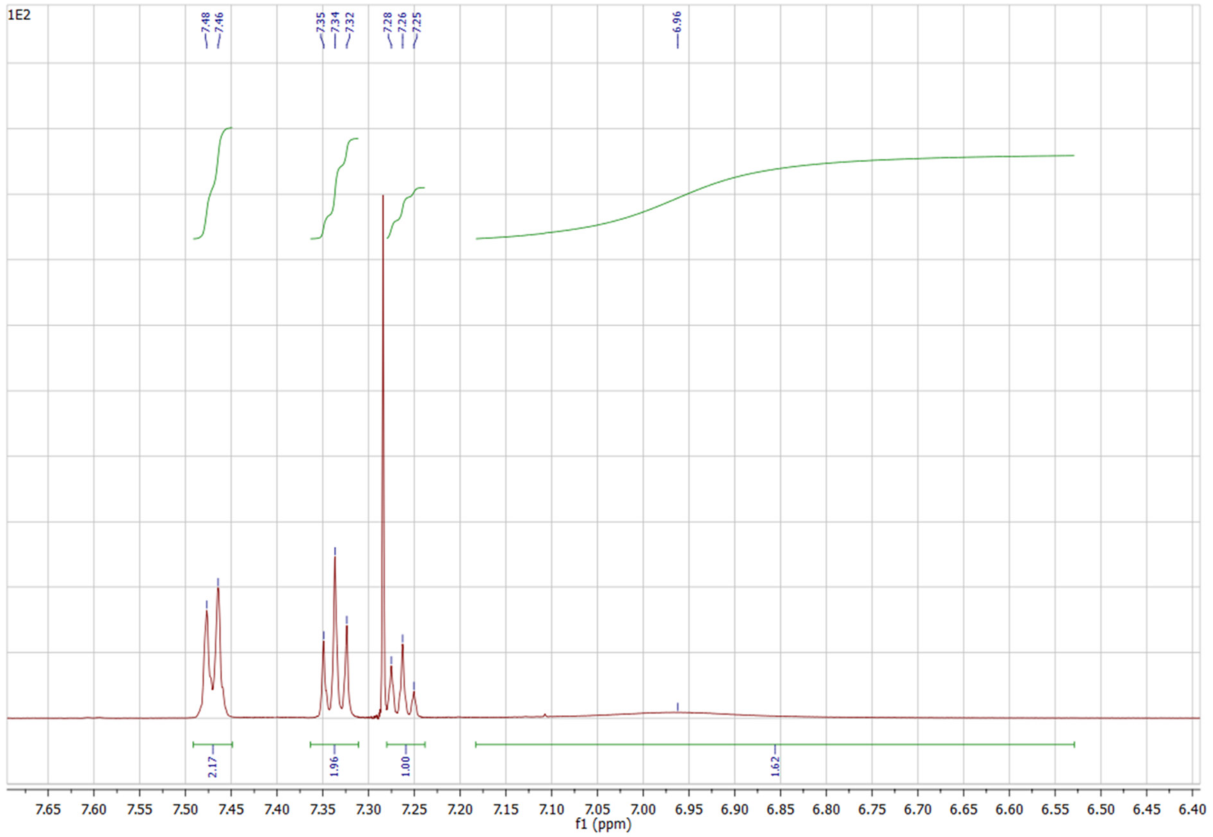

Figure S9-b ^{1}HNMR spectrum of compound **2**

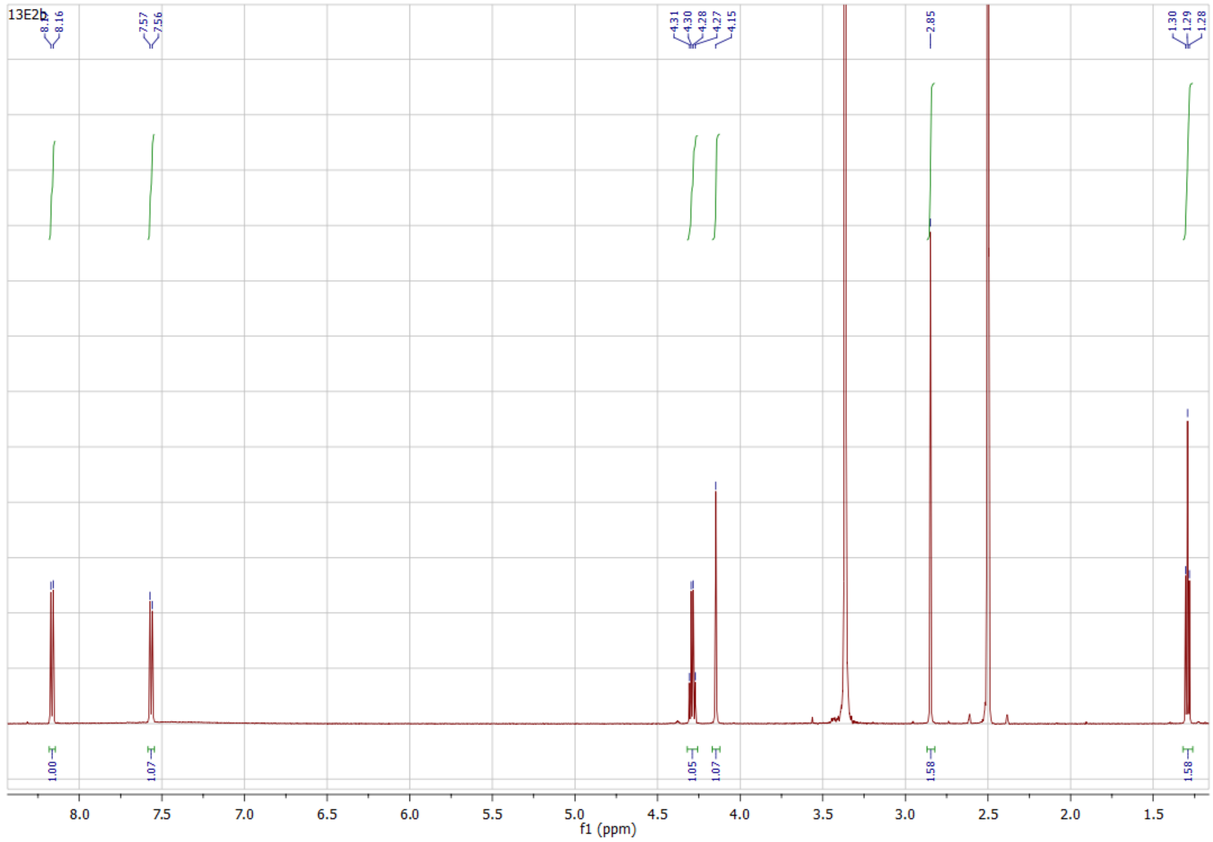

Figure S10-a ^{1}HNMR spectrum of compound **3**

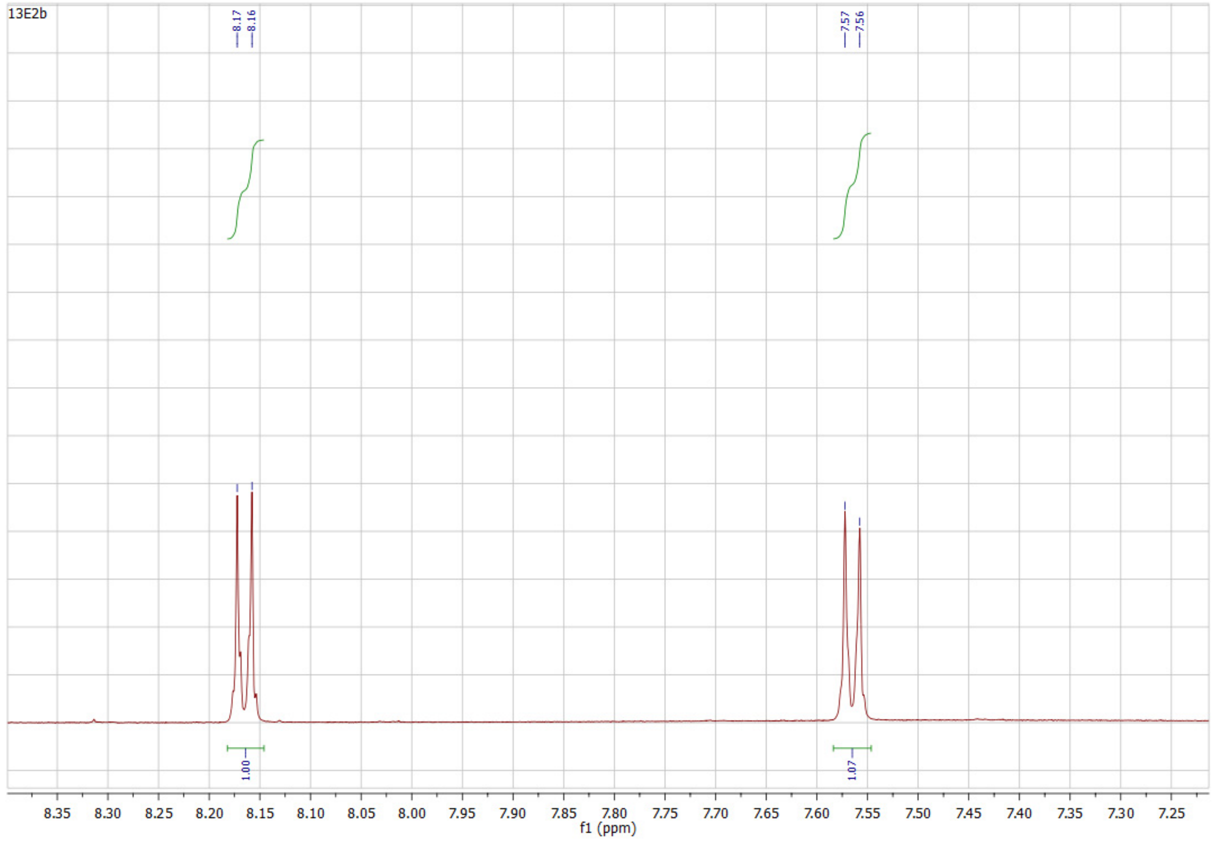

Figure S10-b ^{1}HNMR spectrum of compound 3

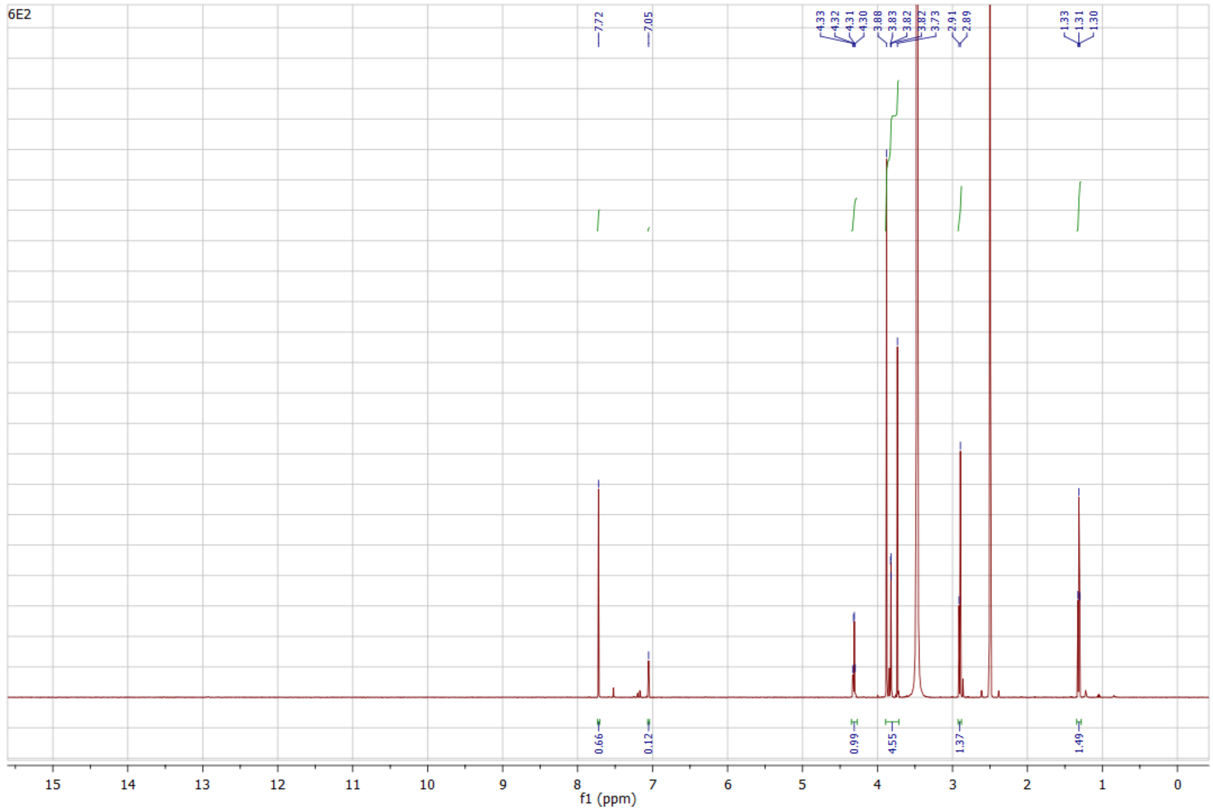

Figure S11-a ^{1}HNMR spectrum of compound **4**

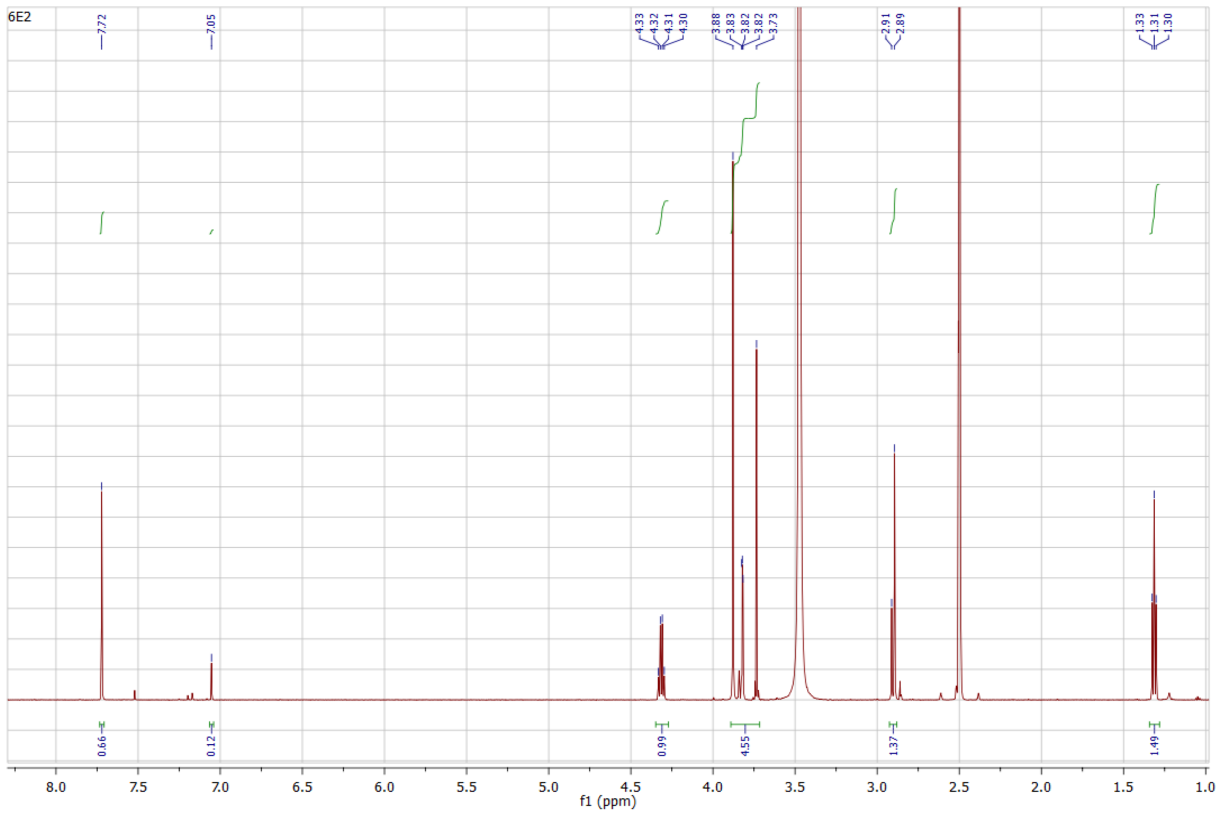

Figure S11-b ^{1}HNMR spectrum of compound **4**

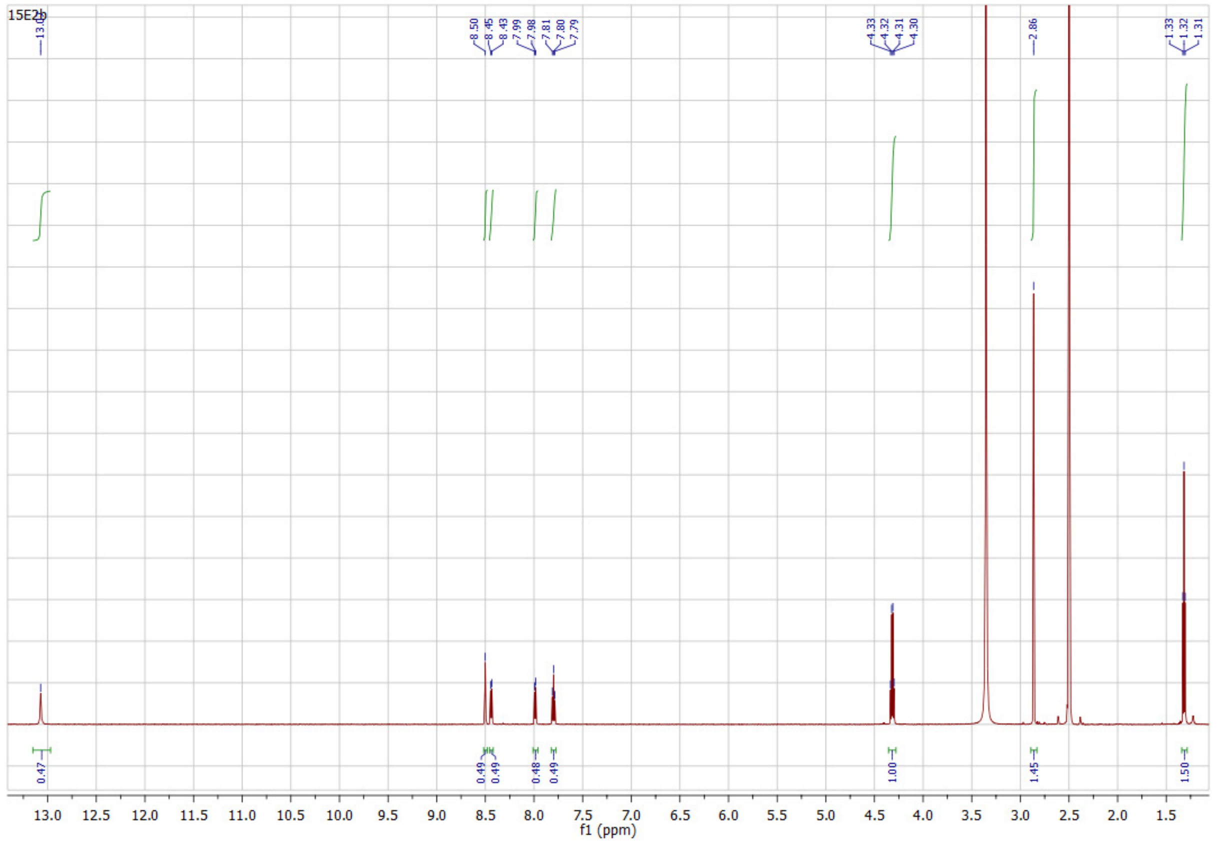

Figure S12-a ^{1}HNMR spectrum of compound **5**

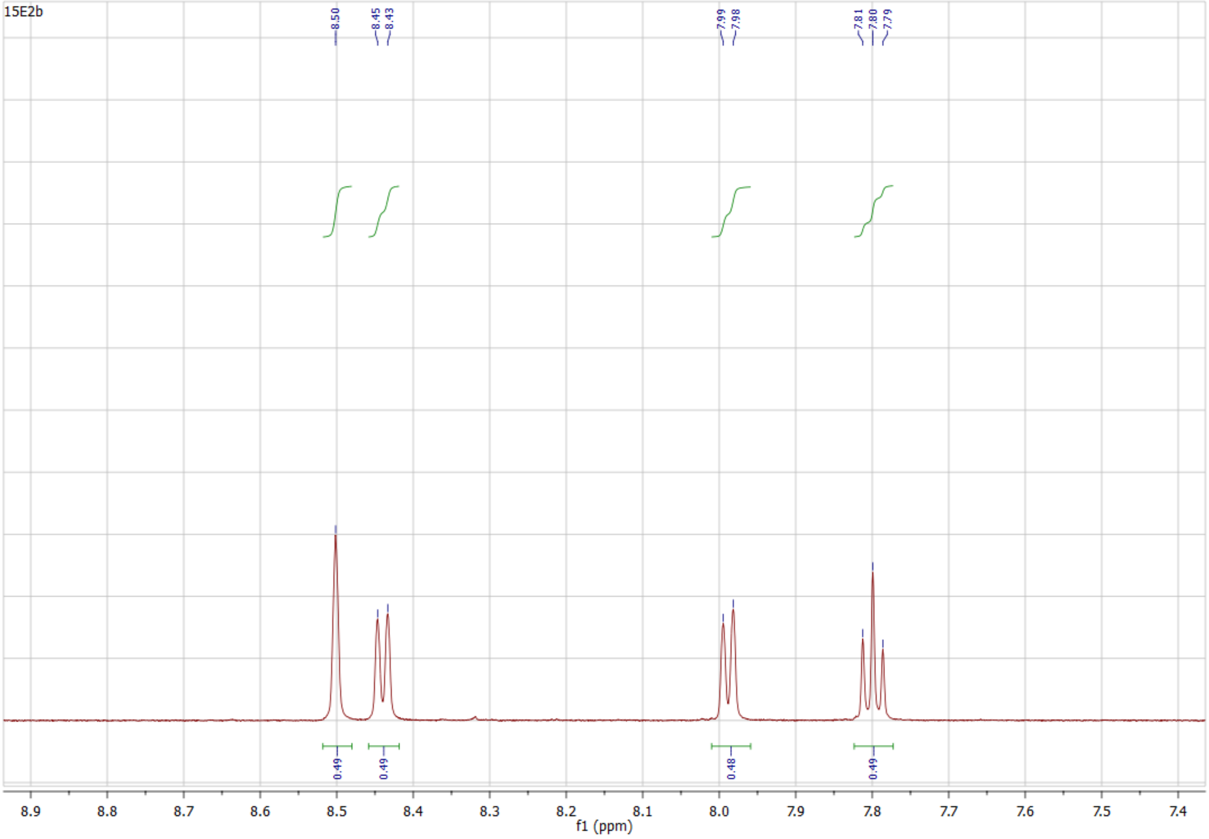

Figure S12-b 1HNMR spectrum of compound **5**

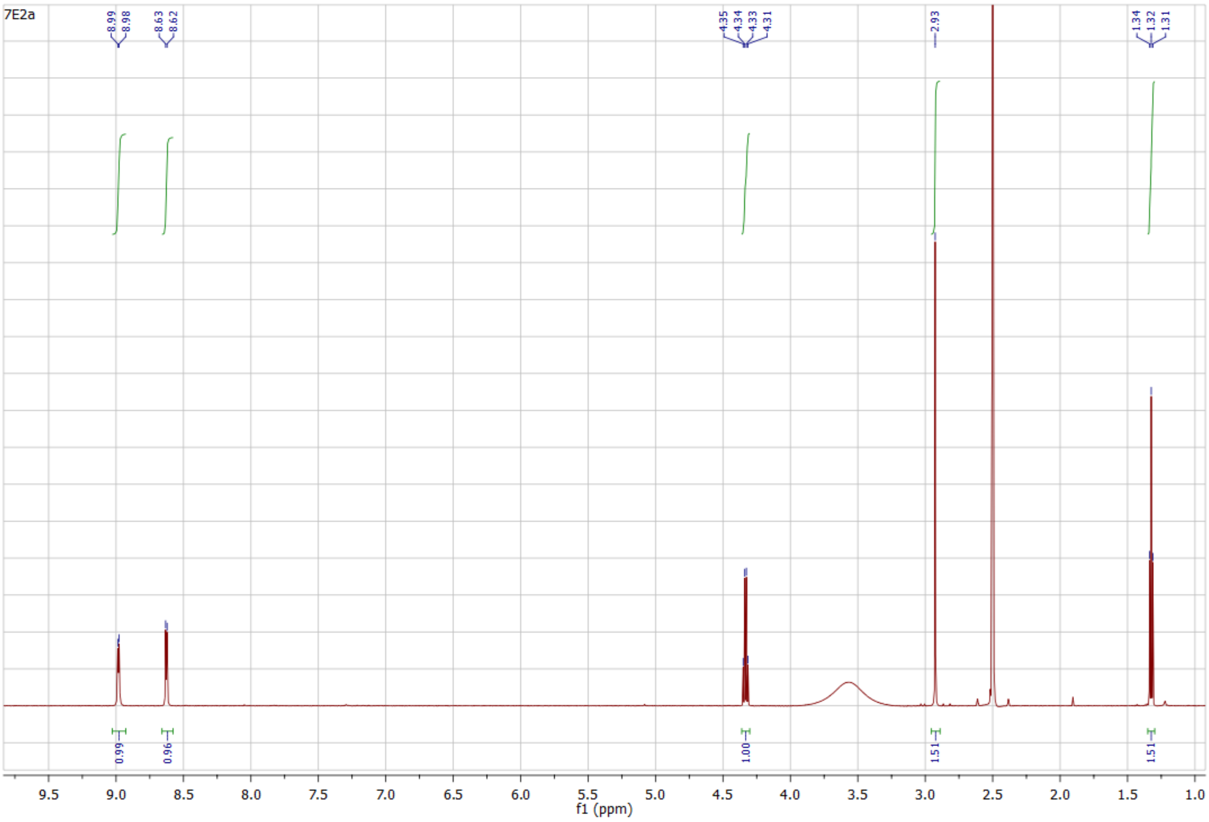

Figure S13-a ^{1}HNMR spectrum of compound **6**

Comp 7

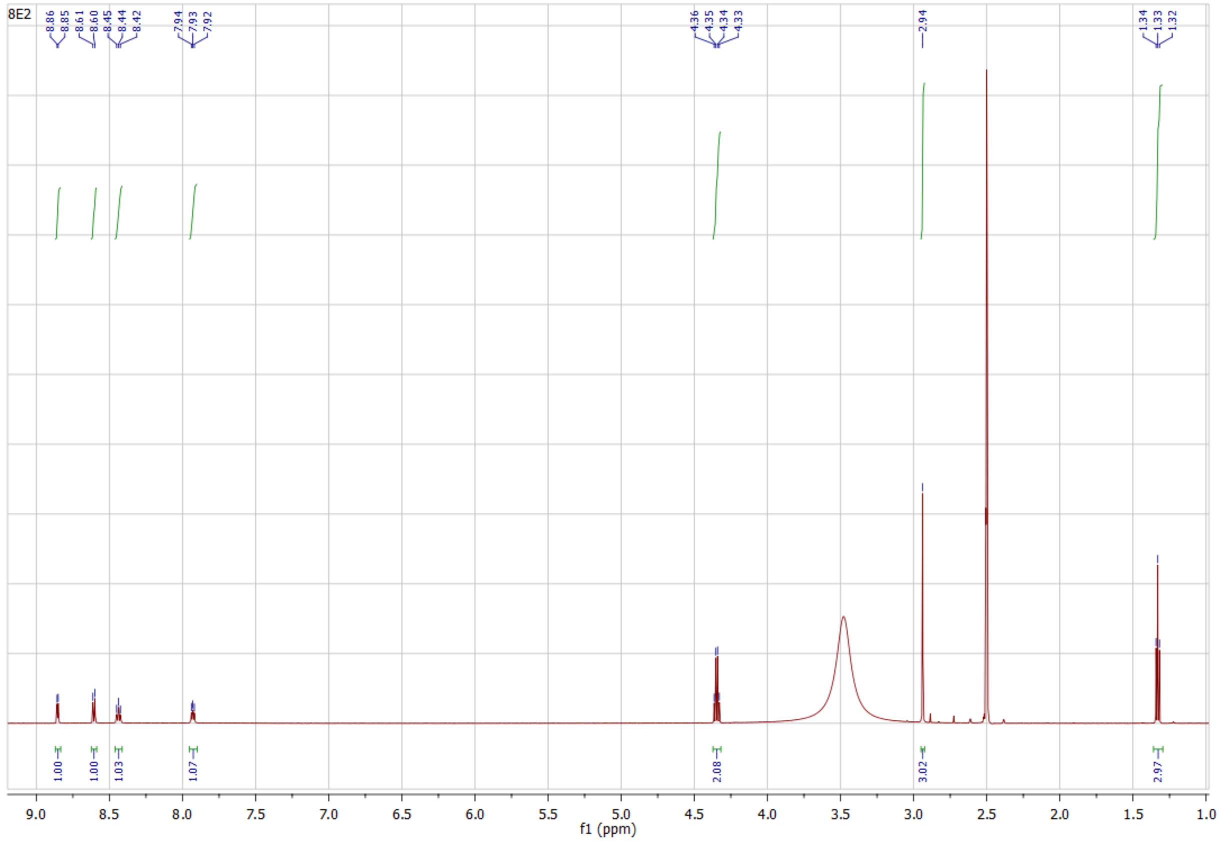

Figure S14-a 1HNMR spectrum of compound **7**

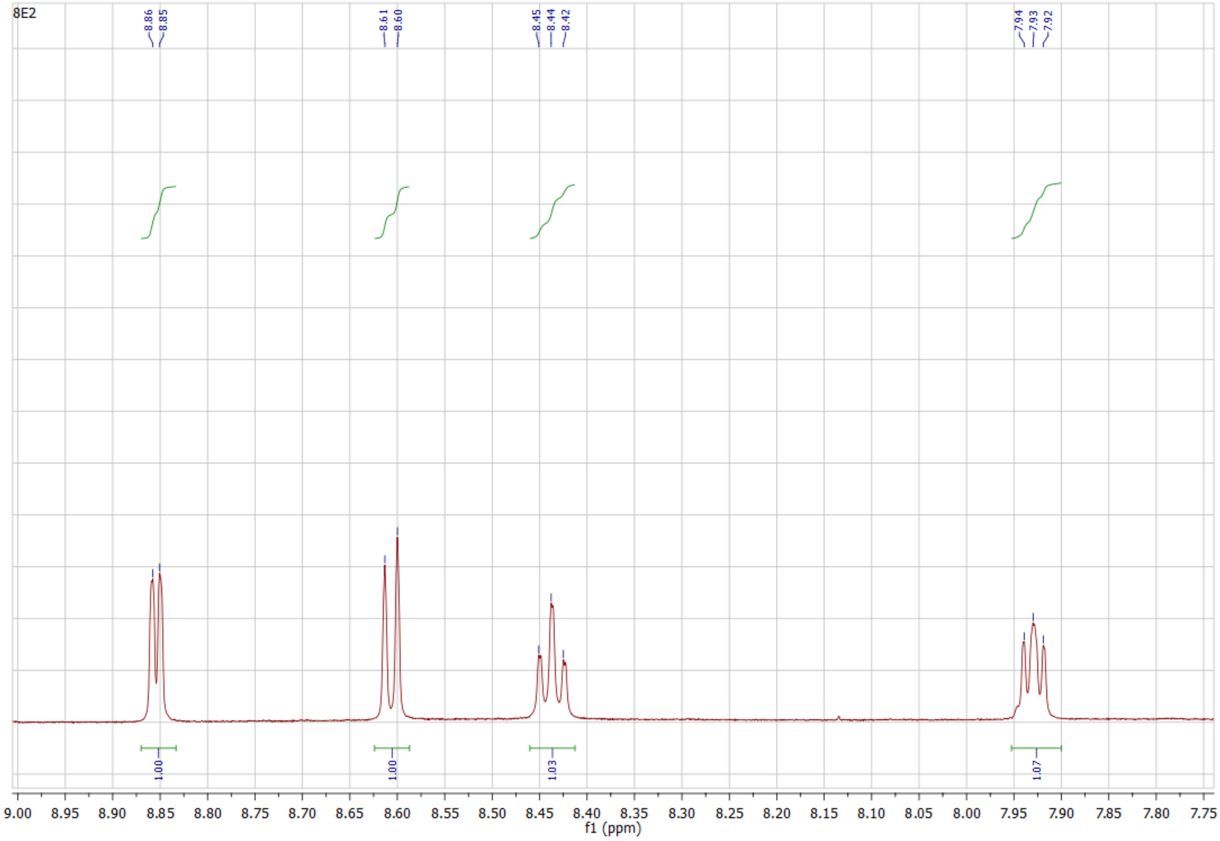

Figure S14-b 1HNMR spectrum of compound **7**

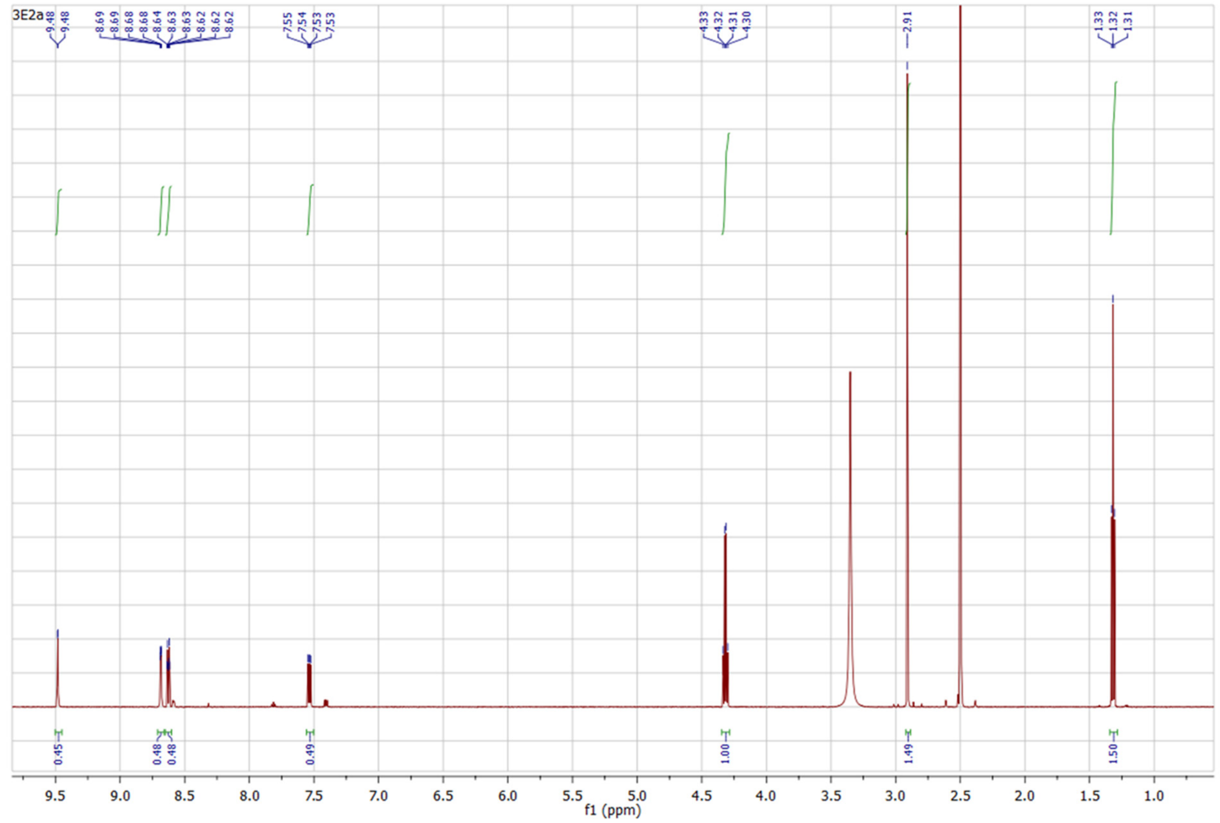

Figure S15-a 1HNMR spectrum of compound **8**

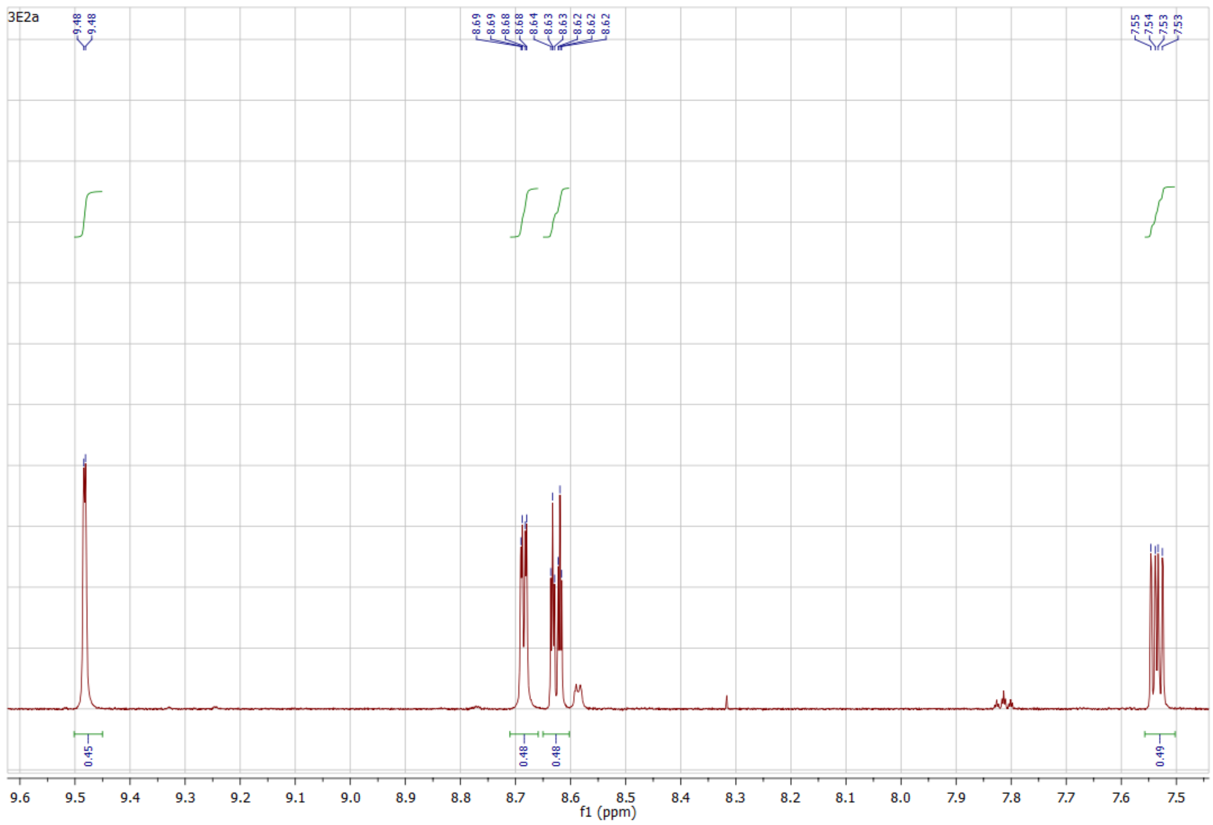

Figure S15-b 1HNMR spectrum of compound **8**
